# Genetic Diversity, Population Structure, Integration of Genome-Wide Association Studies and Machine Learning for Antibacterial Trait Analysis in the Mediterranean Spice Laurel (*Laurus nobilis*)

**DOI:** 10.3390/plants15131997

**Published:** 2026-06-27

**Authors:** Gülşah Karataş, Amjad Ali, Ünal Karık, Muhammad Azhar Nadeem, Muhammad Aasim, Mehmet Bedir, Muhammad Tanveer Altaf, Waqas Liaqat, Sarmad Ali Qureshi, Fawad Ali, Ruziyev Farid, Pablo Federico Cavagnaro, Muhammad Qasim Shahid, Syeid Amjad Ali, Ahmad Alsaleh, Faheem Shehzad Baloch

**Affiliations:** 1 Department of Food Engineering, Tokat Gaziosmanpaşa University, Tokat 60250, Türkiye; gulsah1988kuscuoglu@gmail.com; 2 Faculty of Agricultural Sciences and Technologies, Sivas University of Science and Technology, Sivas 58000, Türkiye; amjadbzu11@gmail.com (A.A.); mehmet.bedir.01@hotmail.com (M.B.); sarmadq95@gmail.com (S.A.Q.); 3 Department of Medicinal Plants, Republic of Türkiye Ministry of Agriculture and Forestry Aegean Agricultural Research Institute, Izmir 35661, Türkiye; unalkarik@gmail.com; 4 Department of Biotechnology, Faculty of Science, Mersin University, Yenişehir, Mersin 33110, Türkiye; azharjoiya22@gmail.com; 5 Department of Biotechnology, Institute of Biochemistry, Sh. Rashidov Samarkand State University, Samarkand 140104, Uzbekistan; 6 Department of Precision Agriculture and Agricultural Robots, Faculty of Agricultural Sciences and Technologies, Sivas University of Science and Technology, Sivas 58000, Türkiye; mshazim@gmail.com; 7 Department of Field Crops, Faculty of Agriculture, Recep Tayyip Erdoğan University, Pazar, Rize 53300, Türkiye; muhammadtanveer.altaf@erdogan.edu.tr (M.T.A.); waqasliaqat0043@gmail.com (W.L.); 8 Department of Plant Resources and Environment, Jeju National University, 102 Jejudaehak-ro, Jeju 63243, Republic of Korea; fawadali365@gmail.com; 9 Department of Genetics, Institute of Biochemistry, Sh. Rashidov Samarkand State University, Samarkand 140104, Uzbekistan; f.ruziyev1985@gmail.com (R.F.); qasim@scau.edu.cn (M.Q.S.); 10 Consejo Nacional de Investigaciones Científicas y Técnicas (CONICET), Ciudad Autónoma de Buenos Aires C1425FQB, Argentina; cavagnaro.pablo@inta.gob.ar; 11 Instituto Nacional de Tecnología Agropecuaria (INTA) Estación Experimental Agropecuaria Mendoza, Luján de Cuyo M5534, Argentina; 12 Department of Plant Biology and Biotechnology, Faculty of Biotechnology and Horticulture, University of Agriculture in Krakow, 31-120 Krakow, Poland; 13 State Key Laboratory for Conservation and Utilization of Subtropical Agro-Bioresources, College of Agriculture, South China Agricultural University, Guangzhou 510642, China; 14 Department of Information Systems and Technologies, Bilkent University, Ankara 06800, Türkiye; syedali@bilkent.edu.tr; 15 Department of Agriculture and Food, Hemp Research Institute, Yozgat Bozok University, Yozgat 66200, Türkiye

**Keywords:** antibacterial activity, GWAS analysis, machine learning, minimum inhibitory concentration, genetic markers, plant-derived bioactive compounds, sustainable production

## Abstract

Laural (*Laurus nobilis*) is a Mediterranean plant with reported antibacterial properties, yet the genetic basis of its antibacterial efficacy remains largely unexplored. This study evaluated the antibacterial activity of *Laurus nobilis* methanolic extracts against *Escherichia coli*, *Staphylococcus aureus*, and *Bacillus cereus*, combined with genome-wide association studies (GWAS) and machine learning (ML) approaches to identify genetic markers and predict antibacterial efficacy in 92 plant samples. Antibacterial tests revealed significant variability in inhibition zones, with *E. coli* showing the highest inhibition (Canakkale2: 24.5 mm), followed by *S. aureus* (Aydin2: 26.0 mm). Minimum inhibitory concentration (MIC) analysis demonstrated notable regional differences; extracts from Mersin3 showed the highest efficacy (MIC = 6.25 mg/mL), while Aydin1 exhibited the lowest activity (MIC = 100 mg/mL). Population structure and neighbor joining tree analysis split the germplasm into two groups. GWAS identified significant genetic markers associated with antibacterial traits, including marker 26557159 for EC-MEAN (*Escherichia coli*-Mean) (*p* = 1.10 × 10^−4^, MarkerR^2^ = 0.1799, genetic variance = 9.41792) and marker 26584774 for BC-MEAN (*Bacillus cereus*-Mean) (*p* = 8.89 × 10^−5^, MarkerR^2^ = 0.18512, genetic variance = 12.48948). Protein–protein interaction network of loci associated with marker trait association (MTA) marker (26557159) indicated involvement in high-affinity secondary active ammonium transmembrane transporter activity, providing insights into genetic regions influencing antibacterial properties. ML models predicted antibacterial activity with high accuracy. XGBoost achieved the best performance for MIC predictions (R^2^ = 0.999, RMSE = 0.434), while random forest (R^2^ = 0.984) demonstrated robust performance for both MIC and disc diffusion assays. LightGBM performed well for MIC prediction (R^2^ = 0.988) but showed limited accuracy for disc diffusion outcomes (R^2^ = 0.695). This study is the first to combine GWAS and ML for predicting antibacterial efficacy in *L. nobilis*, identifying specific genetic markers (e.g., 26557159, 26584774) and demonstrating that XGBoost achieves near-perfect MIC prediction (R^2^ = 0.999). These findings provide a genomic and computational foundation for marker-assisted breeding of laurel with enhanced antibacterial properties and support the sustainable use of plant-derived anti-microbials.

## 1. Introduction

Medicinal and aromatic plants are rich sources of bioactive compounds, including phenols, flavonoids, and tannins. Some compounds in these phenolic subclasses have demonstrated pronounced antibacterial activity against a wide range of plant pathogens [1]. *Laurus nobilis* L. (family Lauraceae), commonly known as laurel or bay leaf, is a Mediterranean aromatic evergreen shrub widely used as a culinary spice and in traditional medicine [2]. The plant produces a diverse array of secondary metabolites, including essential oils (predominantly 1,8-cineole, eugenol, and α-terpinyl acetate), phenolic compounds (such as flavonoids, phenolic acids, and tannins), and sesquiterpene lactones (e.g., costunolide and parthenolide) [3]. Among these, the phenolic and terpenoid fractions have been extensively documented for their anti-microbial, antioxidant, and anti-inflammatory properties, primarily attributed to their ability to disrupt bacterial cell membranes and inhibit microbial enzyme systems [1].

Synthetic agrochemicals have significantly increased agricultural yields. However, their long-term environmental impacts, health risks, and growing pathogen resistance have driven the search for sustainable, eco-friendly alternatives. In this context, plant-derived extracts have gained increasing attention as natural antibacterial agents, with laurel (bay leaf) emerging as a particularly promising candidate due to its broad-spectrum anti-microbial properties [4]. Recent studies have demonstrated strong antibacterial properties in extracts from diverse plant species. For instance, Akinboye et al. [5] reported potent antibacterial activity of ethanol extracts from *Searsia lancea*, with minimum inhibitory concentrations (MICs) ranging from 0.01 to 2.50 mg/mL. Similarly, extracts of two *Oldenlandia* species, *O. corymbosa* and *O. umbellata*, effectively inhibited gram-positive and gram-negative bacteria [6], and metal-complexed extracts of *Combretum microphyllum* exhibited enhanced antibacterial effects against *Escherichia coli*. In addition, strong antibacterial activities were found for methanolic and ethanolic extracts of *Emblica officinalis*, *Acacia nilotica*, and *Carum copticum* against *Xanthomonas* spp. and *Erwinia carotovora* [7]. Similarly, ethanolic extracts of lemongrass (*Cymbopogon citratus*) and bay leaf (*Laurus nobilis*) inhibited lactic bacteria, *Staphylococci*, and *E. coli*, with MIC values in the range of 2.5–10 mg/mL [8]. These studies underscore the substantial antibacterial potential of some plant-derived extracts and support their use as sustainable alternatives to synthetic anti-microbials.

Traditional phenotyping methods for assessing antibacterial activity in plants include disc diffusion assays, broth microdilution for MIC determination, and agar well diffusion tests [9]. While these methods are reliable for direct measurement of antibacterial efficacy, they are time consuming and low throughput and provide no information about the underlying genetic architecture of the observed traits. Beyond conventional phenotypic evaluation, including antibacterial inhibition assays and disease severity assessments, advanced analytical approaches such as genome-wide association studies (GWAS) and machine learning (ML) have emerged as powerful tools for dissecting the genetic and biochemical basis of antibacterial traits in plants. GWAS enables the systematic identification of genetic variants associated with complex phenotypes by correlating genome-wide markers with measured traits [10], thereby facilitating the discovery of loci involved in the biosynthesis of bioactive compounds with antibacterial activity. Complementarily, ML algorithms, such as random forests (RFs), support vector machines, and neural networks, can integrate complex genomic, chemical, and phenotypic datasets to predict antibacterial performance with high accuracy [11]. The combined application of GWAS and ML enables the detection of non-linear relationships and subtle interactions that may remain undetected by conventional statistical approaches [12], while also facilitating the virtual screening of plant-derived compounds and accelerating the discovery of novel antibacterial agents [13].

Despite its advantages, GWAS is sensitive to population structure and linkage disequilibrium, which may produce false marker–trait associations (MTA) if not properly controlled. Therefore, accurate GWAS for disease resistance or antibacterial traits requires dense genome-wide genotyping and careful evaluation of population stratification to identify reliable trait-associated alleles [14,15,16]. Failure to account for population structure can lead to misleading associations arising from uneven allele distributions across subpopulations rather than true genetic effects. In plant microbiology, artificial intelligence (AI)-based tools can facilitate the interpretation of large-scale genomic, proteomic, and transcriptomic datasets, thereby improving understanding of plant–microbe interactions and supporting the development of sustainable disease management strategies [17]. In antibacterial screening, AI-driven approaches have significantly improved the accuracy and efficiency of MIC assessments and disc diffusion assays. Automated image analysis enables precise detection of microbial growth and inhibition zones, thereby reducing observer bias and improving reproducibility [18]. Predictive ML models can forecast MIC values based on plant chemical composition, pathogen characteristics, and environmental variables, supporting the targeted selection of effective antibacterial agents [19]. Similarly, AI-assisted analysis of disc diffusion assays facilitates automated measurement of inhibition zones and prediction of pathogen resistance patterns [20]. ML algorithms such as RF, LightGBM, and XGBoost are particularly effective at handling high-dimensional, non-linear datasets, making them well suited for antibacterial activity prediction and multi-omics data integration [21,22,23].

Despite increasing interest in laurel as a source of bioactive compounds, the antibacterial activity of methanolic extracts against important pathogens, such as *E. coli*, *S. aureus*, and *B. cereus*, remains poorly characterized across diverse germplasm collections. Moreover, no previous study has applied GWAS or ML approaches to identify MTA linked to antibacterial efficacy in laurel, nor have genomic-based predictive models been developed for this species. To date, no candidate genes associated with antibacterial properties of laurel have been reported. The hypothesis of this study was that natural genetic variation within *L. nobilis* populations significantly contributes to differences in antibacterial activity, and that integrating GWAS with ML approaches can effectively identify and predict key genetic determinants underlying these traits. The overall objectives of this study were to evaluate the antibacterial activity of *L. nobilis* methanolic extracts and to elucidate its genetic basis using GWAS and ML approaches by (i) quantifying the antibacterial efficacy of 92 laurel accessions against *E. coli*, *S. aureus*, and *B. cereus* using disc diffusion assays, and against *E. coli* and *S. aureus* using MIC assays; (ii) identifying genetic markers associated with antibacterial traits through GWAS; (iii) constructing protein–protein interaction networks and performing Gene Ontology (GO) enrichment analysis for candidate genes; and (iv) developing and comparing ML models (RF, LightGBM, XGBoost) for predicting antibacterial activity.

## 2. Results

### 2.1. Antibacterial Efficacy of Laurus nobilis

The antibacterial activity of laurel methanolic extracts against three bacterial strains (*E. coli*, *S. aureus*, and *B. cereus*) was evaluated using the disc diffusion method. The results revealed substantial and significant variations in inhibition zones among the laurel accessions (Supplementary Table S1). In addition, significant differences in antibacterial potency were found among the geographical origins (provinces) of the accessions (Table 1). The inhibition zones, measured in millimeters, indicate the anti-microbial efficacy of the *L. nobilis* extracts against each bacterial strain. For *E. coli*, the mean inhibition across all the accessions tested was 14.8 ± 4.2 mm, with a range of 7.0–24.5 mm. The maximum inhibitory effects were found in accessions from Aydin, with a mean inhibitory value of 22.3 mm, followed by accessions from Canakkale (mean: 21.4 mm) and Hatay (mean: 20.8 mm) (Table 1). The individual accessions with the strongest antibacterial effects were Canakkale2 (24.5 mm), Aydin2 (23.5 mm), and Hatay1 (23.0 mm) (Table 1). Conversely, the lowest bacterial inhibitory effects were observed in accessions from Sinop, with a mean of 9.0 mm (Table 2). The accession with the lowest activity was Sinop1 (7.0 mm), followed by Samsun1 (7.5 mm), Mugla4 (8.5 mm), and Sinop3 (8.5 mm) (Supplementary Table S1). Overall, these data reflect geographical variability in the potency of extracts against *E. coli*.

For *S. aureus*, the inhibitory efficacy of the laurel extracts was comparable to that of *E. coli*, with an overall mean of 16.9 ± 4.6 mm, and a range 7.5–26.0 mm. The most effective extracts were from Kahramanmaras (mean: 25 mm), followed by Aydin (24.0 mm), Samsun (21.2 mm), and Zonguldak (21.2 mm). In contrast, accessions from all other provinces had values <20 mm (Table 1). The accessions Aydin2 and Izmir4 demonstrated the highest inhibitory effects, both with 26.0 mm, followed by Kahramanmaras (25.0 mm). Balikesir4, Hatay1, Antalya1, Kocaeli1, Samsun3, and Zonguldak1 also showed strong antibacterial activity, with inhibition zones exceeding 20 mm. In contrast, the accessions from Istanbul had the lowest antibacterial activities, with a mean of 10.9 mm, followed by Mugla (11.3 mm) and Duzce (11.5 mm) (Supplementary Table S1). When considering individual accessions, extracts from Mugla1 (7.5 mm), Sinop2 (8.0 mm), and Hatay1 (8.0 mm) showed the lowest inhibition (Table 1). In general, the *S. aureus*-inhibitory data presented in Table S1 and Table 1 suggest variation in the composition of bioactive compounds of laurel extracts associated with the geographical origin of the accessions, as well as among the individual accessions from a particular provenance.

For *B. cereus*, the laurel extracts revealed a much weaker inhibitory effect than against *E. coli* and *S. aureus*, with an overall mean inhibition zone of 4.4 ± 5.6 mm, and a range of 0.0–17.0 mm (Supplementary Table S1). For this bacterium, the effect of the geographical origin (province) of the accessions was particularly marked. All accessions from 13 provinces showed no antibacterial effects against *B. cereus* (mean inhibition zones: 0.0 mm). In contrast, accessions from Hatay, Balikesir, and Izmir consistently showed the highest antibacterial activity, with mean values of 13.7, 13.3, and 12.4 mm, respectively (Table S1 and Table 1). The strongest antibacterial activity was observed in extracts from Balikesir1, with an inhibition zone of 17.0 mm, followed by Hatay1, Antalya1, and Izmir3, all with mean inhibition zones of 16.0 mm (Supplementary Table S1). These results demonstrate significant variation in the antibacterial properties of extracts from laurel accessions associated with their geographic origins, likely reflecting genetic diversity across plant materials from different regions, as well as adaptations to local environmental conditions and soil compositions. These findings highlight the range of *L. nobilis*’s potential as a natural antibacterial agent.

### 2.2. Minimum Inhibitory Concentration (MIC)

The MIC results provided a comprehensive overview of antibacterial efficacy against *E. coli* and *S. aureus*, with significant variability observed among geographical regions (Table 2). The MIC values of *L. nobilis* extracts against *E. coli* ranged from 6.25 mg/mL (Mersin3) to 100 mg/mL (Aydin1). Extracts from Mugla, Giresun, Samsun, Duzce, Kocaeli, Yalova, Bursa, Izmir, Canakkale, and Istanbul demonstrated lower MIC values (6.25 mg/mL), indicating stronger antibacterial activity. In contrast, the Aydin1 extract exhibited the lowest efficacy, with an MIC of 100 mg/mL. Provinces such as Hatay, Antalya, and Kastamonu generally showed moderate activity, with MIC values of 12.5 mg/mL. Extracts from Zonguldak, Bursa, Kocaeli, Rize, and Balikesir also exhibited relatively higher MIC values (50 mg/mL), reflecting variability in antibacterial potential among samples. For *S. aureus*, MIC values were more consistent and generally lower than those observed for *E. coli*, indicating greater susceptibility to the extracts. MIC values of 3.125 mg/mL and 6.25 mg/mL were most frequently observed, particularly in samples from Mugla, Mersin, Antalya, and Giresun. However, higher MIC values were noted in provinces such as Kocaeli, Bursa, Aydın, and Istanbul, with some samples reaching 25 mg/mL. The findings revealed regional variations in antibacterial efficacy, with *S. aureus* showing greater susceptibility to tested extracts. Potential bioactive hotspots were identified in Mugla, Giresun, and Mersin, emphasizing the importance of regional biodiversity.

### 2.3. Genetic Diversity and Population Structure of Evaluated Germplasm

**Figure 1:**
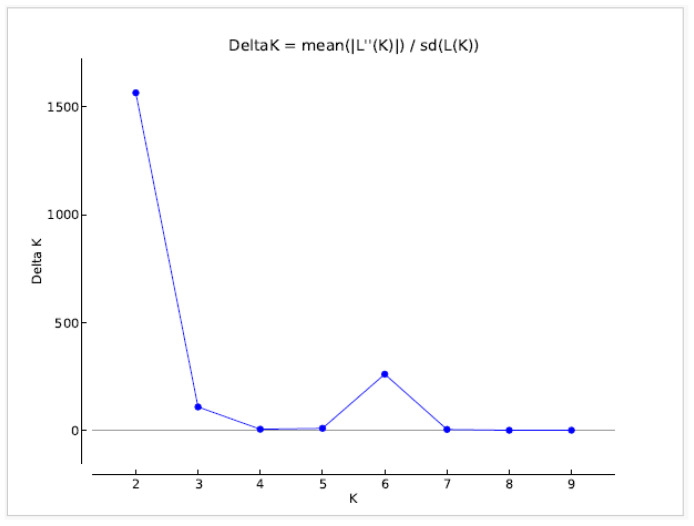
ΔK confirming the population numbers of evaluated germplasm using DArTseq marker system.

**Figure 2:**
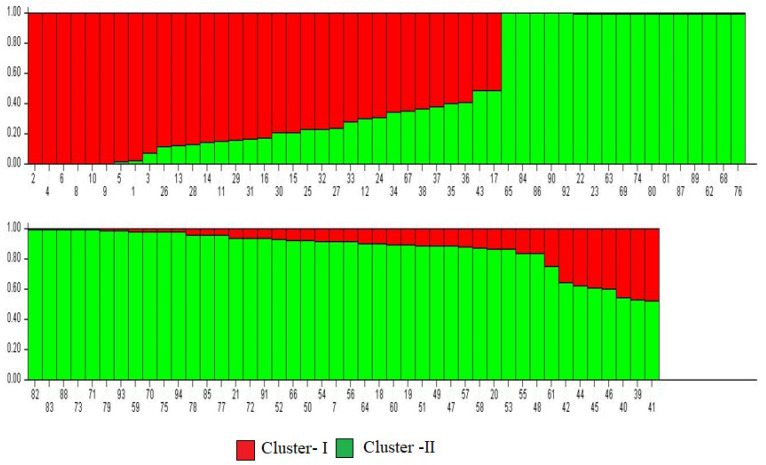
Population structure analysis of laurel germplasm using the DArTseq marker system.

A total of 17.934 DArTseq markers were obtained through GBS analysis as a raw marker data set from Diversity Arrays Technology. By filtering this raw data set (PIC value, call rate, and reproducibility), a total of 12.188 high-quality markers were kept for further analysis. To investigate the genetic relationship between the 94 laurel genotypes, population structure analysis was performed, and the delta K value indicates that the laurel population can be divided into two distinct groups (Figure 1 and Figure 2). Genotypes were assigned to a specific cluster based on a membership coefficient ≥75% (Supplementary Table S2). Genotypes with a membership coefficient lower than 75% were considered an admixture group. Figure 2 of the population structure confirmed the clustering of the evaluated germplasm into two clusters (I and II) based on the defined membership coefficient criteria. Cluster I and Cluster II have a simple genetic structure. Figure 2 reveals the existence of two clusters represented by different colors: red (Cluster-I) and green (Cluster-II). According to the results, Cluster I comprises 22 accessions, while Cluster II comprises 54 accessions. Interestingly, 18 genotypes did not cluster within any population, indicating lower membership coefficients, and were considered an admixture population.

### 2.4. Genetic Diversity and AMOVA Structure Analysis

The germplasm evaluated in this study was collected from four regions of Türkiye (Mediterranean, Black Sea, Aegean, and Marmara regions). Diversity indices were calculated according to the collection regions of 94 genotypes (Table 3). Genotypes from the Black Sea region showed higher effective alleles (1.626), gene diversity (0.529), and expected heterozygosity (0.358). Genotypes from the Mediterranean region showed the minimum effective alleles (1.431), gene diversity (0.380), and expected heterozygosity (0.253). In molecular variance analysis, populations were compared by performing AMOVA. It revealed higher variations between populations (13%) and within populations (87%). AMOVA analysis results are given in Table 4.

### 2.5. Principal Coordinate Analysis

**Figure 3:**
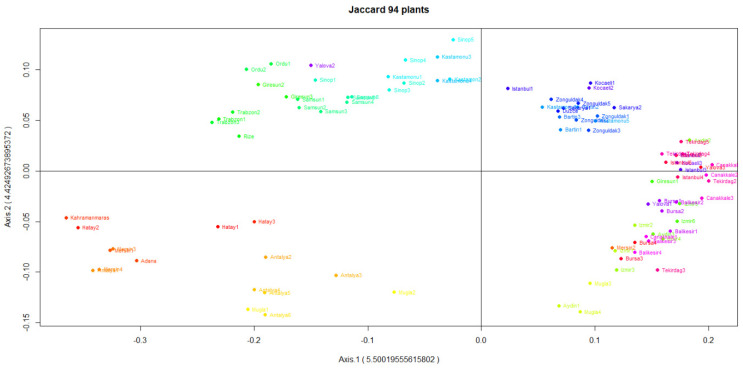
Principal coordinate (PCoA) analysis of 94 laurel genotypes according to city in four geographical regions.

Principal coordinate analysis (PCoA) was performed to visualize the genetic relationships among the 92 laurel genotypes based on DArTseq marker data (Figure 3). The first two principal coordinates explained a substantial proportion of the total genetic variation and revealed a clear pattern of genetic differentiation among the studied genotypes. Genotypes originating from the Mediterranean region were generally positioned separately from most of the Marmara, Aegean, and Black Sea accessions. In contrast, genotypes from the Marmara, Aegean, and Black Sea regions showed partial overlap and formed a broader distribution pattern within the coordinate space. The Black Sea genotypes exhibited the widest dispersion across the plot, indicating a high level of genetic variability within this region. Marmara genotypes also displayed a relatively broad distribution. Conversely, Mediterranean genotypes tended to form a more distinct cluster, with limited overlap with the remaining geographical regions. The Aegean genotypes were distributed between Mediterranean and northern populations, occupying intermediate positions within the coordinate system (Figure 3).

### 2.6. Neighbor-Joining Analysis

**Figure 4:**
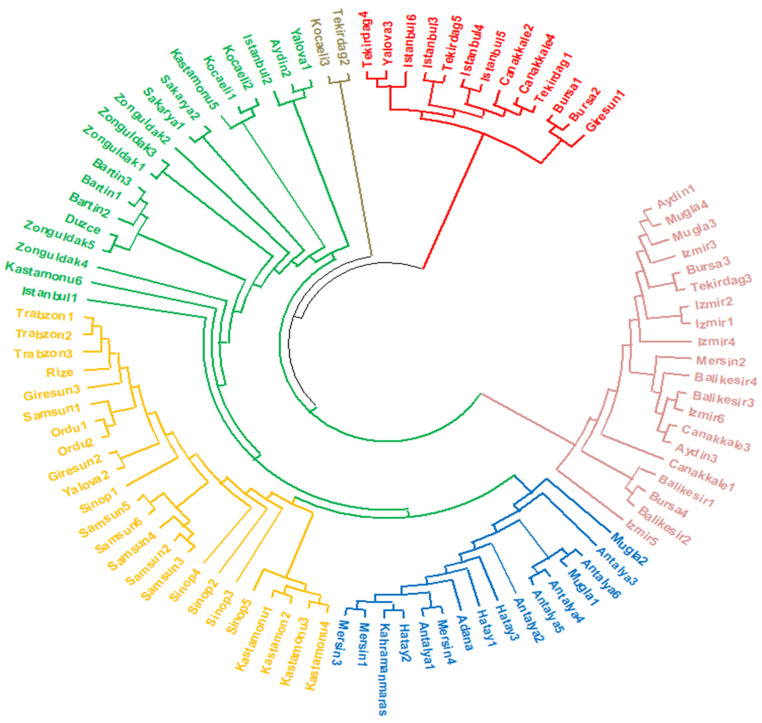
Neighbor-joining clustering of the 94 laurel genotypes using DArTseq marker system.

Neighbor-joining (NJ) analysis divided 94 germplasms into two populations based on their collection areas (Figure 4). NJ clustering supported population structure, with two distinct populations.

### 2.7. Genome-Wide Association Studies

**Figure 5:**
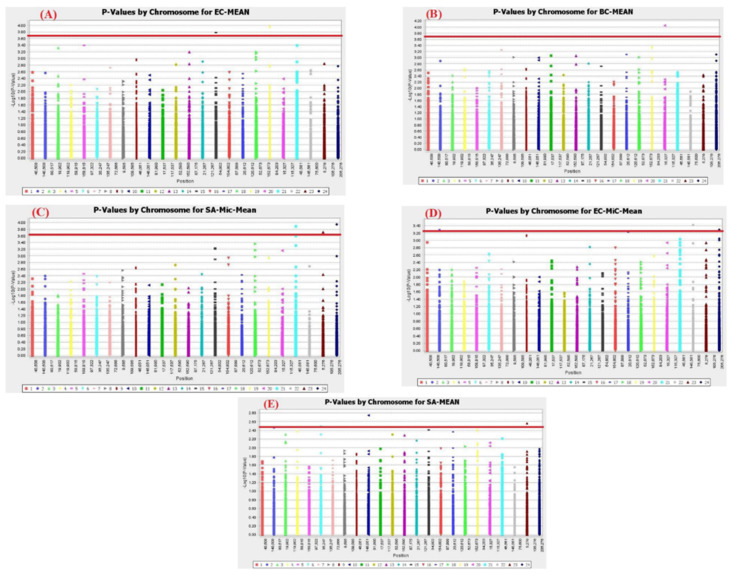
Manhattan plots of genome-wide association study results for antibacterial traits in *Laurus nobilis:* (**A**) *Escherichia coli* mean, (**B**) *Bacillus cereus* mean, (**C**) *Staphylococcus aureus* minimal inhibitory concentration, (**D**) *Escherichia coli* minimal inhibitory concentration, (**E**) *Staphylococcus aureus* mean.

GWAS analysis of 92 *L. nobilis* accessions using the DArTseq platform revealed significant MTS, as summarized in Table 5 and Figure 5. For the EC-MEAN trait, two SNP markers at positions 26,557,159 and 26,574,788 showed strong associations (*p*-values of 1.10 × 10^−4^ and 1.66 × 10^−4^, respectively) and accounted for a substantial proportion of the genetic variation, as indicated by their MarkerR^2^ values of 0.18 and 0.20, respectively. Each of these markers explained 9.4% of the genetic variance (Table 5). For the SA-MEAN trait, marker 26584578 displayed a statistically significant association with a *p*-value of 0.0018 and a moderate MarkerR^2^ of 0.13, while marker 26565222 showed a slightly weaker association (*p* = 0.00275) and a MarkerR^2^ of 0.11. Both markers contributed a relatively small proportion of genetic variance (2.16 × 10^−4^).

Regarding BC-MEAN, marker 26584774 emerged as highly significant, with a *p*-value of 8.89 × 10^−5^ and a robust MarkerR^2^ of 0.19, explaining the highest genetic variance observed in this dataset (12.5%). For the EC-MIC-Mean trait, several markers, including 26584078, 26569339, and 26583551, showed significant associations, with *p*-values ranging from 3.80 × 10^−4^ to 5.24 × 10^−4^ and MarkerR^2^ values of 0.15112, 0.18149, and 0.1585, respectively. These markers collectively explained 54.6% of the genetic variance.

In the case of SA-MIC-Mean, marker 26567210 recorded the lowest *p*-value (1.13 × 10^−4^) and the highest MarkerR^2^ (0.21555) among the SA-associated markers. Additionally, markers 26563218 and 26586695 showed significant associations, with *p*-values of 1.30 × 10^−4^ and 1.95 × 10^−4^ and MarkerR^2^ values of 0.18 and 0.23, respectively. These markers cumulatively accounted for a substantial percentage of the genetic variance (18.8%) for this trait. These findings underscore the successful identification of several genetic markers strongly associated with anti-microbial-activity-related traits in the laurel genotypes.

### 2.8. Candidate Gene Identification, Protein–Protein Interaction (PPI) Network, and Gene Ontology (GO) Enrichment

**Figure 6:**
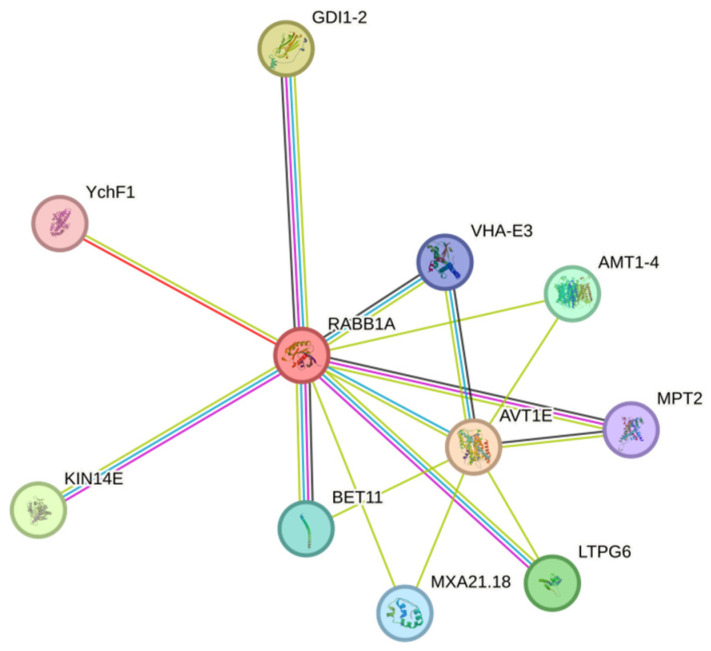
Protein–protein interaction network of loci associated with marker 26557159, generated using STRING, highlighting RAB1A and its interacting proteins.

**Figure 7:**
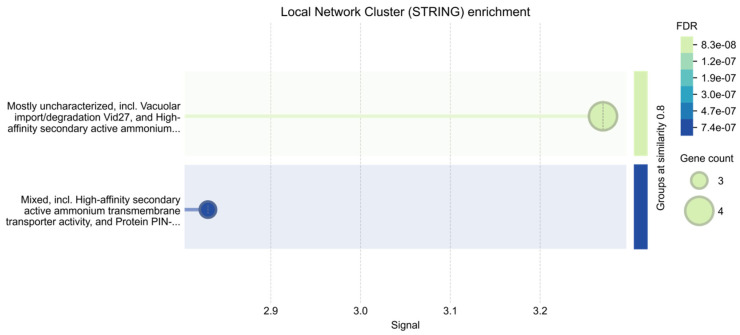
GO enrichment analysis of *Arabidopsis* orthologs of loci associated with marker 26557159, revealing their associated functions.

BLAST analysis of significant MTAs identified a single *Arabidopsis* ortholog for each marker under stringent thresholds. PPI networks were constructed to identify potential interacting partners of identified orthologs. The marker-associated locus formed a protein interaction cluster consisting of 11 proteins (Figure 6). GO enrichment analysis of this cluster (Figure 7) revealed significant enrichment for high-affinity secondary active ammonium transmembrane transporter activity, indicating a coordinated role in nitrogen homeostasis or stress response.

### 2.9. Prediction of Antibacterial Activity Using Machine Learning Models

The results indicated that ML models exhibited variable performance across different metrics and traits (Table 6). RF demonstrated strong predictive power for both MIC and disc diffusion assays, explaining nearly 97% of the variance in MIC data. Its low RMSE and MAE indicated consistent prediction accuracy. RF’s ability to capture small-scale differences in MIC values and its proximity to actual values further emphasized its precision. In disc diffusion, RF demonstrated even better performance, explaining 98% of the variance and yielding lower RMSE and MAE values. Its superior ability to minimize prediction errors in disc diffusion outcomes confirmed its robustness and adaptability.

LightGBM also showed strong performance in MIC predictions, with an R^2^ of 0.988, accounting for 99% of the variance in MIC values. Its RMSE (1.315) was lower than that of RF, indicating smaller prediction errors, and its MAE (0.304) was low, indicating high accuracy. However, LightGBM underperformed in disc diffusion predictions, explaining only 69% of the variance. The RMSE (4.045) and MAE (3.273) were relatively high, with an MSLE of 0.414 and MedAE of 2.740, suggesting limited suitability for disc diffusion data.

XGBoost achieved the highest performance in MIC predictions, explaining nearly 100% of the variance in data. It recorded an R^2^ value of 0.999, with minimal prediction errors and a low MAE. The MSLE value of 0.003 indicated very small differences in MIC prediction. XGBoost maintained consistent accuracy across the dataset, making it the most effective model for MIC predictions. For disc diffusion, XGBoost explained 78% of the variance (R^2^ = 0.781), demonstrating good predictive performance, although lower than that observed for MIC. XGBoost was identified as the most effective model for predicting antibacterial efficacy based on MIC values, achieving near-perfect R^2^ values and the lowest error metrics. RF demonstrated balanced performance across both assays, highlighting its versatility, whereas LightGBM excelled at MIC prediction but showed limitations in disc diffusion analysis. These findings underscore the importance of selecting appropriate models based on the dataset characteristics and assay type.

## 3. Discussion

Higher MIC values were associated with greater efficacy against *S. aureus* across provinces. Kavitha and Satish [7] evaluated the antibacterial properties of nine medicinal plant extracts, including *Emblica officinalis*, *Acacia nilotica*, and *Carum copticum*. Using methanol and ethanol as solvents, these extracts exhibited significant inhibitory effects against Xanthomonas campestris pv. vesicatoria and other pathogens. Additional species such as *Pedalium murex* and *Hyptis suaveolens* also showed activity, underscoring solvent specificity in antibacterial activity. Divya et al. [6] analyzed petroleum ether, ethanol, and aqueous extracts of *Oldenlandia corymbosa* and *Oldenlandia umbellata*. Petroleum ether and ethanolic extracts effectively inhibited both gram-negative and gram-positive bacteria (e.g., *Pseudomonas aeruginosa* and *B. subtilis*) even at low concentrations (56.25 µg/mL). These results highlight the versatility of *Oldenlandia* species against diverse pathogens. Abebe et al. [24] tested crude aqueous and Co(II)-complex extracts of *Combretum microphyllum*. The Co(II)-complex showed potent antibacterial activity, with the highest inhibition (3.5 mm) against *E. coli*. In contrast, the crude aqueous extract showed no activity, indicating that metal complexes significantly enhance bioactivity. Ibrahim and Kebede [25] studied extracts of *Moringa oleifera*, *Azadirachta indica*, and *Lepidium sativum*. Methanol-based extracts showed higher antibacterial activity than aqueous extracts. Notably, heat treatment at 55 °C abolished the anti-microbial activity, confirming the thermal sensitivity of the bioactive compounds.

Akinboye et al. [5] examined ethanol and acetone extracts of *Antidesma venosum* and *Searsia lancea*. The ethanol extract of *Searsia lancea* showed the lowest MIC range (0.01–0.57 mg/mL) among the tested samples, suggesting its potential to develop potent antibacterial agents against *Streptococcus species* and *E. coli*. Karimou et al. [8] reported anti-microbial activities of aqueous and ethanolic extracts of *C. citratus* and *L. nobilis*. Ethanolic bay leaf extracts showed the highest inhibition zones (21.4 mm) against lactic bacteria, followed by significant efficacy against *E. coli* and *staphylococci*, with MIC values ranging from 2.5 to 10 mg/mL. Ramos et al. [26] showed that hydro-distillation of laurel leaves provided superior anti-microbial efficacy, particularly against *Brochothrix thermosphacta* and *Shewanella putrefaciens*. Cold aqueous extraction showed limited activity, indicating that essential oils outperform other extraction methods. Ozcan et al. [27] found methanolic seed extracts of *L. nobilis* to be highly effective against *Haemophilus influenzae* (15.33 mm inhibition zone). This activity was significantly higher than that of leaf essential oils, highlighting seeds as a potent source of antibacterial compounds. Al-Bayati [28] reported that essential oils and methanol extracts from *Thymus vulgaris* and *Pimpinella anisum* seeds exhibited robust antibacterial activity. MIC values ranged from 15.6 µg/mL (*T. vulgaris*) to 500 µg/mL, with the strongest effects against *Staphylococcus aureus* and *B. cereus*. Liu et al. [29] demonstrated that ethanolic olive leaf extracts inhibited the growth of *Listeria monocytogenes* and *Salmonella enteritidis* by 100% at 62.5 mg/mL, with *E. coli* showing 95% inhibition. This finding confirms its broad-spectrum efficacy. Coccimiglio et al. [30] emphasized the inhibitory properties of oregano extracts against gram-negative and gram-positive bacteria, with MIC values ranging from 6.3 to 25 µg/mL. These results validate oregano’s potential as a natural preservative and anti-microbial agent. Singh et al. [31] evaluated various plant extracts, including *Morinda citrifolia* and *Syzygium aromaticum*. Noni (*M. citrifolia*) showed the highest inhibition zone (>14 mm), followed by garlic and clove. MIC values as low as 1.25 mg/mL against multiple pathogens highlight the utility of diverse plants in anti-microbial formulations. Xedzro et al. [32] identified clove and negro pepper as the most effective spices against *Listeria monocytogenes* and methicillin-resistant *S. aureus* (MRSA). MIC values ranged from 0.05% to 0.4%, suggesting high potency for clinical and food applications.

Recent studies on *L. nobilis* support our observation that antibacterial efficacy varies with eco-geographic origin, plant part, and extraction chemistry. Essential oils from wild laurel collected across seven altitudinal locations in central-southern Italy displayed clear chemotypic shifts (e.g., 1,8-cineole, methyl-eugenol, α-terpinyl acetate, linalool) as characterized by chemometrics [33]. Bojović et al. [34] reported strong in vitro activity of laurel leaf and fruit essential oils against gram-positive bacteria (*S. aureus*, *E. faecalis*, *B. subtilis*) and Candida. Solvent and plant-part effects have also been documented in Turkey, where 70% ethanolic extracts of leaf and fruit showed anti-microbial and anti-quorum-sensing potential [35]. Other comparisons of ethanol/water extracts have reported marked differences in bioactivity profiles [36]. Additional mechanistic and translational studies have highlighted the antibiofilm and antibacterial activity of laurel oils, along with in silico target interactions [37], as well as potential synergy between laurel essential oil (and its dominant 1,8-cineole chemotype) and gentamicin against MRSA. A recent synthesis on Lebanese bay leaves further emphasizes that altitude, climate, and harvest stage can shape chemotypes and associated pharmacological activity [4].

Based on 12,188 high-quality DArTseq markers, population structure analysis divided the 92 Turkish bay laurel genotypes into two primary genetic clusters. Meanwhile, 18 genotypes were identified as admixed due to membership coefficients falling below 75%. These findings reveal a clear, yet not fully isolated, genetic architecture within Turkish bay laurel germplasm. The two main clusters indicate regional genetic differentiation, whereas the admixed cohort suggests historical or ongoing gene flow between populations. This pattern of population structuring aligns with previous research on Turkish laurel germplasm using iPBS-retrotransposon markers, which found that STRUCTURE, PCoA, and NJ analyses clustered genotypes primarily by collection region and province [38]. Furthermore, Yılmaz and Çiftçi [39] observed high levels of polymorphism and substantial genetic diversity using ISSR and SCoT markers, reinforcing the premise that Turkish laurel germplasm possesses a diverse genetic foundation, ideal for conservation and breeding initiatives.

Cluster I was predominantly composed of genotypes sampled from the Mediterranean region, specifically Hatay, Kahramanmaraş, Mersin, Adana, and Antalya, as well as several accessions from the eastern Black Sea, including Trabzon, Ordu, Giresun, Samsun, and Rize. The grouping of genotypes from Hatay, Mersin, Adana, Antalya, and Kahramanmaraş carries biological relevance, given that these provinces fall within or are directly adjacent to the natural Mediterranean habitat of bay laurel. This aligns with Karık et al. [38], who identified the Mediterranean region as harboring the highest genetic diversity in Turkish laurel and proposed it as a key diversity center for the species. Consequently, Cluster I likely denotes a primary Mediterranean gene pool for Turkish bay laurel. The presence of multiple eastern Black Sea genotypes within this cluster can potentially be attributed to historical gene flow, seed dispersal, and anthropogenic transport of botanical material.

Cluster II predominantly featured genotypes from the Marmara, western Black Sea, and western coastal provinces, including Istanbul, Tekirdag, Canakkale, Bursa, Balikesir, Sakarya, Kocaeli, Bartin, Zonguldak, Duzce, Kastamonu, Izmir, Aydin, and Yalova. A significant proportion of these genotypes showed high membership coefficients, suggesting a relatively homogeneous genetic background. This specific clustering pattern can likely be linked to geographical continuity, comparable ecological conditions, and localized adaptation. In a similar way, Karık et al. [38] documented that Marmara, Black Sea, and Aegean genotypes clustered heavily within the same population, whereas Mediterranean genotypes formed a distinct group. Furthermore, Yılmaz and Çiftçi [39] underscored that Turkish laurel genotypes exhibit robust molecular polymorphism and coherent genetic alliances aligned with their geographic origins. Consequently, the Cluster II map represents a second major gene pool forged by regional connectivity, local adaptation, and the circulation of propagating material among adjacent provinces.

The admixed group comprised genotypes with membership coefficients falling short of the 75% threshold, featuring several accessions from Samsun, Sinop, Kastamonu, Mugla, Antalya, Yalova, and various transitional zones. The identification of these admixed genotypes represents a pivotal finding of this research, as it demonstrates that the two primary clusters do not exist in complete isolation. Rather, these individuals harbor genetic inputs from both lineages. This configuration may stem from pollen-mediated gene flow, seed dispersal, hybridization, or the anthropogenic transport of botanical material. In a comparable study, Rego et al. [40] documented elevated levels of genetic admixture within wild Laurus azorica populations using SSR markers, underscoring that gene flow exerts a powerful influence on the population dynamics of Laurus species. Consequently, the admixed genotypes uncovered here likely serve as transitional populations bridging the Mediterranean, Aegean, Marmara, and Black Sea gene pools. These genotypes hold significant value because they may harbor novel allelic combinations derived from divergent genetic backgrounds.

The NJ analysis divided the 92 bay laurel genotypes into two major groups, largely consistent with the population structure results obtained from the Bayesian STRUCTURE analysis. The first NJ group predominantly consisted of genotypes originating in the Mediterranean region, including Hatay, Kahramanmaraş, Mersin, Adana, and Antalya, as well as several accessions from the eastern Black Sea region. The close clustering of these genotypes suggests that they share a relatively common genetic background despite their geographical separation. Bay laurel is considered a native component of Mediterranean ecosystems, and the Mediterranean region has previously been identified as the most genetically diverse area for Turkish laurel populations [38]. Therefore, the grouping of these accessions may reflect a shared ancestral gene pool that has been maintained through historical gene flow and long-term natural distribution. The presence of eastern Black Sea genotypes within this group further suggests that genetic exchange among distant populations has occurred over time, probably facilitated by pollen dispersal, seed movement, and centuries of human utilization of the species.

The second NJ group was mainly composed of genotypes collected from the Marmara, western Black Sea, and western coastal regions, including Istanbul, Tekirdag, Bursa, Canakkale, Balikesir, Kocaeli, Sakarya, Bartin, Zonguldak, Duzce, and Kastamonu. The clustering of these accessions indicates a higher degree of genetic similarity among populations from northern and northwestern Turkey. Geographic proximity, similar ecological conditions, and regional adaptation may have contributed to the formation of this distinct genetic group. In addition, the extensive exchange of plant material among neighboring provinces may have promoted genetic homogenization within this cluster. Similar regional clustering patterns have been reported in other perennial Mediterranean tree species, where both environmental adaptation and historical dispersal routes contribute to the formation of geographically structured populations. The NJ analysis confirmed the existence of two principal genetic lineages within Turkish bay laurel germplasm while simultaneously revealing substantial genetic connectivity among populations. The consistency between NJ and STRUCTURE analyses strengthens the reliability of the observed population structure and suggests that both historical biogeography and contemporary gene flow have contributed to shaping the current genetic landscape of Turkish bay laurel. These findings highlight the importance of conserving representatives from both major genetic groups and admixed genotypes to preserve the full spectrum of genetic diversity available for future breeding and conservation programs.

The PCoA results demonstrated that Turkish bay laurel germplasm possesses a structured genetic organization while maintaining substantial connectivity among populations. The overall clustering pattern was highly consistent with the STRUCTURE and NJ, indicating that the observed genetic relationships are robust and biologically meaningful. One of the most notable findings of the present study was the relative separation of Mediterranean genotypes from most accessions originating from the Marmara, Aegean, and Black Sea regions. This observation suggests the existence of a distinct Mediterranean genetic background within Turkish bay laurel germplasm. Karık et al. [38] similarly reported that Mediterranean populations exhibited the highest genetic diversity among Turkish laurel populations and formed a relatively separate genetic group. Since the Mediterranean Basin represents the natural distribution area and evolutionary center of *L. nobilis*, the differentiation of Mediterranean genotypes may reflect both long-term adaptation and the retention of ancestral genetic variation. Maintaining this distinct gene pool is particularly important for future conservation and breeding efforts, as it may harbor unique alleles associated with adaptation to Mediterranean environmental conditions. In contrast, the considerable overlap observed among Aegean, Marmara, and Black Sea genotypes suggests extensive historical and contemporary gene flow among these regions. Such overlap is commonly observed in outcrossing woody species, where pollen dispersal, seed movement, and human-mediated transport contribute to genetic connectivity among populations. Yılmaz and Çiftçi [39] reported high genetic diversity and substantial genetic relationships among Turkish laurel genotypes, indicating that regional populations are not genetically isolated. Likewise, Rego et al. [40] demonstrated extensive admixture in Laurus populations and emphasized that gene flow represents a major evolutionary force shaping genetic structure within the genus.

The position of the Aegean genotypes deserves particular attention. Unlike Mediterranean accessions, which tended to cluster more distinctly, Aegean genotypes were distributed between Mediterranean and northern populations. This pattern suggests that the Aegean region may function as a biogeographical transition zone connecting different genetic lineages. Similar transition-zone effects have been reported in several Mediterranean tree species, where historical migration routes, climatic gradients, and human activities have facilitated the mixing of previously differentiated gene pools. The intermediate positioning of Aegean accessions therefore supports the hypothesis that this region has played an important role in facilitating gene exchange among Turkish bay laurel populations. Another important observation was the broad dispersion of Black Sea genotypes across the coordinate space. This distribution indicates substantial within-region diversity and suggests that Black Sea populations are genetically heterogeneous. The extensive coastal distribution of bay laurel throughout the Black Sea region, combined with local environmental variation and long-term human utilization, may have contributed to the accumulation of diverse genetic backgrounds within this region. Similar patterns of high within-region diversity have been reported in perennial woody species characterized by broad ecological amplitudes and long evolutionary histories.

The NJ dendrogram further supports this interpretation. Genotypes originating from the same province were frequently assigned to different clusters and subclusters. For example, accessions from Mugla, Antalya, Istanbul, Sinop, Giresun, and Zonguldak occurred in different branches of the dendrogram. Such a pattern indicates that genetic relationships are not determined solely by geographical origin. Instead, historical dispersal, pollen-mediated gene flow, seed exchange, cultivation practices, and human-mediated movement of plant material have likely contributed to the current population structure. Similar observations were reported by Karık et al. [38], who concluded that Turkish laurel populations exhibit extensive admixture and regional overlap despite showing evidence of geographical differentiation. The PCoA, STRUCTURE, and NJ analyses consistently indicate that Turkish bay laurel germplasm is characterized by two major genetic backgrounds accompanied by substantial admixture. The Mediterranean populations appear relatively distinct, whereas Aegean, Marmara, and Black Sea populations remain genetically interconnected. These findings suggest that the combined effects of geographical differentiation, historical migration, ecological adaptation, and extensive gene flow have shaped the current genetic architecture of Turkish bay laurel.

The GWAS analysis of 92 *L. nobilis* genotypes identified several significant genetic markers associated with key traits such as EC-MEAN, SA-MEAN, and BC-MEAN and highlighted potential targets for marker-assisted breeding. For instance, markers 26557159 and 26574788 were strongly linked to the EC-MEAN trait, exhibiting significant associations with MarkerR^2^ values of 0.1799 and 0.19752, respectively. These findings are consistent with recent studies on other crops that used high-throughput techniques to identify genetic markers associated with antioxidant properties. Megha et al. [41] used a 62K SNP genic chip to identify markers associated with antioxidant properties in pigeon pea genotypes. Similarly, in our study, markers such as 26584078 and 26569339 were associated with EC-MIC-MEAN, demonstrating the value of GWAS in identifying loci linked to oxidative stress responses. These findings underscore the importance of markers such as 26557159, 26574788, and others in explaining genetic variation in antioxidant-related traits, thereby enhancing marker-assisted selection. Furthermore, the study by Nadeem et al. [42] on common beans also utilized DArTseq markers, identifying DArT-3369938 as highly associated with antioxidant activity, explaining 14.61% of the trait variance. This is comparable to the marker 26584774 in our study, which accounted for 12.48948% of genetic variance in BC-MEAN. Both studies highlight the utility of DArTseq markers for understanding antioxidant traits and their potential for use in breeding programs to improve crop resilience and nutritional quality.

Habyarimana et al. [43] also identified several QTLs linked to antioxidant properties in sorghum landraces, including a pleiotropic major-effect marker, Chr1_61095994, which explained 27–31% of the variance. Similarly, in our study, marker 26567210, which showed a significant association with SA-Mic-Mean, explained a substantial portion of the variance, underscoring the importance of identifying high-effect markers to enhance antioxidant traits in breeding programs. The findings of Yadav et al. [44] in pearl millet, which identified 218 SNPs associated with antioxidant traits, further validate the importance of SNP-based studies. Our study also identified novel loci with significant associations, such as 26567210, highlighting the potential of such markers for breeding antioxidant-rich crops. Ro et al. [45] identified key loci for phenolic content and antioxidant activity in eggplant, demonstrating the broad applicability of MTA across species. The identification of antioxidant hotspots on chromosomes 11 and 12 in our study further reinforces this concept, as these regions are linked to key traits such as flavonoids and γ-oryzanol. Our GWAS results, along with findings from the studies of Megha et al. [41], Nadeem et al. [42], Habyarimana et al. [43], Yadav et al. [44], and Ro et al. [45], demonstrate the potential of using genetic markers to improve antioxidant traits in crops. These markers, especially those with high MarkerR^2^ values and significant genetic variance contributions, represent valuable tools for marker-assisted breeding aimed at improving the nutritional and health-promoting properties of *L. nobilis* and other crops. Recent GWAS efforts in other crops continue to resolve the polygenic control of phenolics, flavonoids, and antioxidant activity and to nominate candidate genes and loci for marker-assisted selection [46,47].

BLAST analysis identified marker 26557159 as linked to the Arabidopsis ortholog AT4G17160 (encoding RABB1A), which was subsequently used for PPI network construction and GO enrichment analysis (Figure 6). The analyses revealed that this marker is located within a functional cluster associated with largely uncharacterized biological processes, including vacuolar import and degradation (e.g., Vid27), high-affinity secondary active ammonium transmembrane transporter activity, and PIN-LIKES (PILS) family members (PILS1/3/4/5/7) involved in intracellular auxin transport (Figure 7). These associations suggest coordinated functions in cellular trafficking, nutrient transport, and hormone homeostasis pathways that are critical for stress adaptation but not fully elucidated [48,49]. Beyond algorithm selection, the quality and standardization of anti-microbial phenotypes are major determinants of model performance. Recent AI-assisted anti-microbial susceptibility testing (AST) pipelines have enabled automated and reproducible measurement of inhibition zones from disc diffusion images [50]. In addition, recent studies highlight the rapid development of AI-driven AST platforms integrating imaging, microfluidics, and multi-omics datasets [51]. Such approaches may reduce inter-operator variability in disc diffusion assays and improve downstream genomic and ML-based predictions.

Comparing multiple ML models, such as RF, LightGBM, and XGBoost offers significant advantages in predicting anti-microbial activity [52]. Each model operates on different algorithms and assumptions, offering unique strengths and weaknesses. Researchers can select the most accurate and robust model for their specific task by evaluating models like RF, which excels at handling noisy and complex datasets [53], whereas XGBoost offers better accuracy when dealing with non-linear relationships [54]. Comparing models helps identify the one that generalizes better to new, unseen data, ensuring predictions can be applied reliably to new samples [55]. Using multiple models reduces bias in model selection and allows researchers to evaluate performance across metrics like R^2^, RMSE, and MAE, providing a comprehensive understanding of the data’s complexity. In some cases, hybrid approaches or model stacking can result in even better accuracy [56]. Thus, comparing multiple ML models leads to more informed decisions, improved generalization, and enhanced accuracy in anti-microbial activity predictions [57,58].

The study examines the performance of ML models in predicting antibacterial efficacy based on MIC and disc diffusion assays. RF showed strong predictive performance, explaining a significant portion of the variance in both MIC and disc diffusion data. Its robustness and versatility make RF a reliable model across diverse datasets. LightGBM performed well in MIC predictions but faced challenges in disc diffusion assessments, suggesting it is least productive model for tasks involving multiple assay types. XGBoost achieved the highest performance in MIC prediction; however, its predictive ability for disc diffusion data was comparatively limited. This highlights the importance of careful model selection in ML-based biomedical applications. No single model consistently outperformed the others across all assay types, indicating that dataset characteristics and prediction objectives should guide model choice. The findings advocate for an informed model selection process that maximizes predictive accuracy and applicability across varied research scenarios. Given the assay-dependent performance observed here, future work may benefit from assay-specific calibration and from incorporating standardized (or automated) zone measurements to reduce label noise in disc diffusion datasets [50].

## 4. Materials and Methods

### 4.1. Plant Material

In this study, plant material was obtained from the Laurel Gene Garden, which was established from seeds of laurel accessions collected from 92 locations across the Mediterranean, Aegean, Marmara, and Black Sea regions of Türkiye. Samples were collected from the Laurel Gene Garden at the Aegean Agricultural Research Institute, an affiliated institute of the General Directorate of Agricultural Research and Policies of the Ministry of Agriculture and Forestry, and were properly labeled. Passport information of the collected laurel populations is provided in Supplementary Table S3. The collected leaves were dried at 35 °C for 72 h and stored in polypropylene bags until further analysis.

### 4.2. Preparation of Methanolic Extracts

One gram of the powdered sample, ground using a grinder (Sinbo, SCM2934, Ömerli, Türkiye), was extracted with 20 mL of an 80% methanol–water solution in a shaking water bath (Microtest, Istanbul, Türkiye) for 150 min. The solvent phase was separated after centrifugation at 7000 rpm for 15 min) (Hermle Z327K, Germany). The remaining solid residue was re-extracted twice under the same conditions. The combined extracts were filtered through Whatman No. 1 filter paper. The filtrates were dehydrated at 60 °C for 48 h and stored at −18 °C until analysis.

### 4.3. Test Organisms

*Staphylococcus aureus* (ATCC 25923), *Escherichia coli* (ATCC 25922), and *Bacillus cereus* (ATCC 11778) used in the microbiological analyses were obtained from Microbiology Laboratory, Department of Food Engineering, Faculty of Engineering and Architecture, Tokat Gaziosmanpaşa University. Prior to the analyses, *S. aureus*, *B. cereus,* and *E. coli* were activated by growing them twice in tryptic soy broth (TSB) medium for 18–24 h at 37 ± 2 °C.

### 4.4. Antibacterial Activity

The disc diffusion method [59] and the microdilution procedure using 96-well plates [60] were employed to determine antibacterial activity. The agar disc diffusion method was used to determine the antibacterial activity of *L. nobilis* methanolic extracts. Initially, *S. aureus*, *B. cereus,* and *E. coli* were cultured on tryptic soy agar (TSA) at 37 °C for 24 h. Subsequently, 0.1 mL of the bacterial suspensions was spread evenly on the surface of the TSA plates, which were then allowed to dry. One colony from each bacterial strain was transferred aseptically into 9 mL of sterile peptone water and homogenized. Bacterial suspensions were collected using sterile swabs, inoculated onto TSA, and left at room temperature for 10 min. Absorbent discs (Whatman disc No. 3, 6 mm in diameter) were impregnated with methanolic *L. nobilis* extracts and placed on the surface of the inoculated plates. Methanol-impregnated discs were used as positive controls. After incubation at 37 °C for 24 h, inhibition zones were measured and expressed in millimeters [61].

### 4.5. Determination of Minimum Inhibitory Concentration (MIC)

The microdilution method in 96-well plates was used to determine the MIC, defined as the lowest concentration of extracts capable of inhibiting bacterial growth. Concentrations of *L. nobilis* extracts in methanol were prepared at the following ratios: 1:1 (100 mg/mL), 1:2 (50 mg/mL), 1:4 (25 mg/mL), 1:8 (12.5 mg/mL), 1:16 (6.25 mg/mL), 1:32 (3.125 mg/mL), 1:64 (1.5625 mg/mL), 1:128 (0.78 mg/mL), 1:256 (0.39 mg/mL), and 1:512 (0.195 mg/mL), respectively. A volume of 100 μL of extracts was added to each microwell. After a 10% dilution was added to the first well, dilutions were transferred sequentially up to the 10th well. No laurel extract was added to the last well, which was considered as the positive control. Subsequently, 90 μL of tryptic soy agar medium and 10 μL of bacterial cultures (*E. coli* and *S. aureus*) were added to each well, followed by incubation at 37 °C for 24 h. At the end of the incubation period, wells showing turbidity, membrane formation, or surface sedimentation were considered potential positives and evaluated for bacterial growth.

### 4.6. Identification of Antibacterial Properties of Bay Leaf by Genome-Wide Associated Studies

#### 4.6.1. DNA Isolation

DNA isolation and determinations of DNA quality and quantity were performed as reported previously by Baloch et al. [62]. DNA was isolated from bay leaves according to the procedure of Doyle and Doyle [63]. Briefly, 1 mL of extraction buffer (2% cetyl trimethylammonium bromide (CTAB); 100 mM Tris-HCl, pH 7.5; 1.4 M NaCl; 20 mM EDTA, pH 8.0; 20 mL/mL β- mercaptoethanol; 20 mg PVP) was added to 100 mg of bay leaf tissue followed by incubation at 60 °C for 1 h. Then, 600 µL of chloroform was added, and the samples were centrifuged. The samples were then washed with 70% ethanol, dissolved in distilled water and treated with RNAase A enzyme.

#### 4.6.2. DNA Quality and Quantity Adjustment

DNA integrity and quantity were estimated based on electrophoresis on 0.8% agarose gels stained with ethidium bromide and visualized under UV light [64]. The samples were also analyzed by means of spectrophotometry, and DNA purity was determined based on the A260/280 nm absorbance ratio [63]. For DArTseq analysis, 20 μL of DNA at a concentration of 50 ng/μL was used. The DNA samples were electrophoresed on 1% agarose gel containing 1.5% TAE buffer at 80 V for 1–1.5 h.

#### 4.6.3. DArTseq-Based Genotyping by Sequencing Analysis

DArTseq technology integrates a complexity reduction technique with NGS platforms [65], enabling the targeted selection of genome regions linked to key plant traits [66]. DNA samples from the 92 *L. nobilis* accessions were sent to Diversity Arrays Technology Pty, Ltd., Bruce, Australia (http://www.diversityarrays.com/), for DArTseq™ analysis, a sequencing-based diversity array technology for SNP marker detection. While often referred to broadly as genotyping-by-sequencing (GBS), DArTseq employs a specific complexity-reduction method using the PstI and MseI restriction enzymes, followed by next-generation sequencing (NGS) on an Illumina HiSeq 2000 platform. This approach enables genome-wide discovery of SNP and SilicoDArT markers.

For optimizing DArTseq in *L. nobilis*, genome fraction selection and representation size were adjusted accordingly. The complexity reduction employed PstI-MseI enzymes, and DNA samples underwent digestion/ligation reactions as described by Kilian et al. [65]. Mixed fragments (PstI–MseI) were amplified using 30 PCR cycles with the following thermal profile: (I) 94 °C for 1 min, (II) 94 °C for 20 s, (III) ramp 2.4 °C/s to 58 °C, (IV) 58 °C for 30 s, (V) ramp 2.4 °C/s to 72 °C, (VI) 72 °C for 45 s, (VII) repeat steps 2–6 for 29 cycles, (VIII) 72 °C for 7 min, (IX) hold at 10 °C [30]. Amplified products from each sample in a 96-well microtiter plate were pooled equimolarly, and then processed on a c-Bot (Illumina, San Diego, CA, USA) for bridge PCR, followed by sequencing on an Illumina HiSeq2000 platform. Sequencing involved 77 single-read cycles. Sequence data were processed using DarT’s proprietary analytical pipelines [66], starting with filtering of FASTQ files to eliminate low-quality sequences. Approximately 4,000,000 sequences per sample were analyzed for marker calling. Identical sequences were condensed into “FASTQcall files” for secondary analysis using DArTsoft14 software, which identified SNP and SilicoDArT (presence/absence of restriction fragments) markers.

### 4.7. Bioinformatic Analysis

#### 4.7.1. Marker-Trait Associations, PPI Networks, and GO Enrichment

Genotyping by sequencing (GBS) analysis resulted in a total of 17934 DArTseq markers. It was very important to screen for the most informative markers for bioinformatics analysis. Keeping this in view, the DArTseq markers were screened according to different parameters such as call rate, polymorphism information content (PIC), and reproducibility. Using these parameters, a total of 12,188 markers were selected, having 100% reproducibility, call rate = 0.9–1, and PIC value = 0.1–0.5. To investigate the level of genetic variation in the 94 laurel genotypes, various parameters such as effective allele number (Ne), observed allele numbers (Na), unbiased expected heterozygosity (uHe), gene diversity (h), Shannon information index (I), and Nei’s genetic distance were measured for four regions by GenAlEx 6.5 software. NJ analysis and principal coordinate analysis (PCoA) were calculated in R. Moreover, GenAlEx 6.5 software was used to perform an analysis of molecular variance (AMOVA). The software STRUCTURE (Version 2.3.4) was employed to investigate the population structure of the germplasm studied, as described by Pritchard et al. [67]. The optimal number of clusters (K subpopulations) was determined by analyzing the data three times using the methodology described by Evanno et al. [68]. The range of clusters considered was set from 1 to 10. Ten independent runs were conducted for each K value, with each run initiating with a burn-in period of 50,000 and 100,000 Markov chain Monte Carlo (MCMC) iterations. Subsequently, the evaluated results were submitted to the Structure Selector (https://lmme.ac.cn/StructureSelector/) to determine the optimal K value.

During this study, GWAS analysis was performed through TASSEL 5.0.5 (https://tassel.bitbucket.io) software for the marker–trait association (MTA) investigation. For the GWAS analysis, the phenotypic data, the filtered marker data set, and the Q-metrics resulting from the structure analysis were used to identify significant MTAs for the traits studied. Moreover, the kinship (K) matrix reported by Bradbury et al. [69] was analyzed using TASSEL 5.0.5 (https://tassel.bitbucket.io). Finally, a mixed linear model (MLM, Q + K) was used to identify loci associated with the studied traits in the evaluated germplasm using TASSEL 5.0.5 (https://tassel.bitbucket.io).

Marker sequences were BLAST-searched against the *Arabidopsis thaliana* genome (TAIR10) using TAIR BLAST 2.9.0+ [70]. Loci exhibiting the highest hit scores and an E-value ≤ 0.003 were selected as orthologs. These Arabidopsis orthologs were queried in STRING (version 12.0) [71], to construct protein–protein interaction (PPI) networks, applying a minimum interaction score of 0.7. Network reliability was enhanced by filtering interactions with a combined score ≥400 [72]. Genes encoding interacting proteins were then subjected to Gene Ontology (GO) enrichment analysis for biological processes using Fisher’s exact test with Bonferroni correction (*p* < 0.05) [73].

#### 4.7.2. Machine Learning Analysis

Machine learning models, namely random forest, LightGBM, and extreme gradient boosting (XGBoost), were employed to predict antibacterial activity based on MIC and disc diffusion assay data. These algorithms were selected because they can model complex non-linear relationships and interactions among predictor variables, which are frequently encountered in biological and microbiological datasets.

Random forest is an ensemble learning method that combines predictions from multiple decision trees constructed using bootstrap samples of the training data and randomly selected subsets of predictor variables [74]. By aggregating the outputs of numerous trees, RF reduces model variance and improves generalization performance while maintaining robustness against overfitting. In addition, RF can effectively handle datasets with correlated variables and complex feature interactions, making it well suited for biological prediction tasks.

LightGBM is a gradient boosting framework that uses a histogram-based algorithm and a leaf-wise tree growth strategy to improve computational efficiency and predictive accuracy [75]. Compared with conventional boosting approaches, LightGBM requires less memory and shorter training times while maintaining strong predictive performance. These characteristics make it particularly useful for datasets containing variables with heterogeneous distributions and non-linear relationships.

XGBoost is an optimized gradient boosting algorithm that sequentially constructs decision trees to minimize prediction error through gradient-based optimization [76]. The method incorporates regularization, shrinkage, and feature subsampling techniques to improve model generalization and reduce the risk of overfitting. Owing to its ability to capture complex non-linear patterns and higher-order interactions, XGBoost has become one of the most widely used algorithms in predictive modeling applications.

Model performance was assessed using leave-one-out cross-validation (LOOCV). Under this validation scheme, a single observation was retained for testing while the remaining observations were used for model training. The procedure was repeated iteratively until every observation had served once as the validation sample [77]. Because each model is trained on nearly the entire dataset during every iteration, LOOCV provides an efficient use of limited experimental data and yields a reliable estimate of predictive performance.

All data preprocessing, model development, hyperparameter tuning, and validation procedures were implemented using custom Python (Version 3.14.0) scripts. The workflow ensured consistent model training and evaluation across all algorithms, with predictions generated independently for each LOOCV iteration before calculating the final performance metrics.

#### 4.7.3. Performance Metrics

Performance metrics were used to assess the accuracy and robustness of ML models for predicting antibacterial activity, specifically MIC and disc diffusion assay outcomes. Each metric provided a different perspective on model performance, ensuring a comprehensive assessment of prediction quality [78,79].

## 5. Conclusions

This study demonstrates that *Laurus nobilis* methanolic extracts possess significant, geographically variable antibacterial activity against *E. coli*, *S. aureus*, and *B. cereus*. GWAS identified genetic markers (e.g., 26557159, 26584774) associated with these traits, with marker 26584774 explaining 12.5% of genetic variance for BC-MEAN. Among the ML models tested, XGBoost achieved near-perfect MIC prediction (R^2^ = 0.999), outperforming random forest (R^2^ = 0.968) and LightGBM (R^2^ = 0.988). These findings provide a genomic and computational foundation for marker-assisted breeding of laurel with enhanced antibacterial properties.

## Figures and Tables

**Table 1:** Variation in antibacterial activity of laurel methanolic extracts according to province of origin.

	Inhibition of *E. coli*	Inhibition of *S. aureus*	Inhibition of *B. cereus*
Province	Mean ± SD	Mean ± SD	Mean ± SD
Antalya	13.8 ± 2.6	19.0 ± 3.0	7.7 ± 6.5
Aydin	22.3 ± 1.0	24.0 ± 2.3	9.2 ± 4.1
Balikesir	13.0 ± 2.4	19.1 ± 1.7	13.3 ± 3.3
Bartin	16.5 ± 1.8	13.7 ± 1.4	1.3 ± 1.2
Bursa	12.3 ± 2.3	15.1 ± 3.9	0.0 ± 0.0
Canakkale	21.4 ± 2.1	19.9 ± 3.3	7.0 ± 1.2
Duzce	19.5 ± 0.5	11.5 ± 0.5	0.0 ± 0.0
Giresun	14.8 ± 2.8	18.1 ± 1.8	8.8 ± 4.5
Hatay	20.8 ± 1.9	12.5 ± 3.9	13.7 ± 2.1
Istanbul	14.0 ± 3.8	10.9 ± 1.5	0.0 ± 0.0
Izmir	16.0 ± 4.3	18.8 ± 6.6	12.4 ± 2.9
Kahramanmaras	17.5 ± 0.5	25.0 ± 1.0	0.0 ± 0.0
Kastamonu	12.5 ± 1.2	16.3 ± 1.4	0.0 ± 0.0
Kocaeli	17.0 ± 2.6	16.3 ± 1.4	0.0 ± 0.0
Mersin	15.4 ± 2.1	14.8 ± 3.1	11.5 ± 1.3
Mugla	17.5 ± 6.3	11.3 ± 3.7	1.5 ± 3.0
Ordu	14.5 ± 0.7	15.3 ± 0.4	0.0 ± 0.0
Rize	18.0 ± 0.0	14.5 ± 0.5	0.0 ± 0.0
Sakarya	17.5 ± 0.7	13.3 ± 1.8	7.5 ± 0.7
Samsun	12.8 ± 2.9	21.2 ± 3.0	2.9 ± 3.4
Sinop	9.0 ± 1.7	13.4 ± 3.0	0.0 ± 0.0
Tekirdag	11.6 ± 2.5	19.0 ± 3.7	0.0 ± 0.0
Trabzon	18.0 ± 4.8	19.2 ± 2.0	0.0 ± 0.0
Yalova	15.2 ± 4.5	14.3 ± 3.2	0.0 ± 0.0
Zonguldak	12.0 ± 1.3	21.2 ± 3.1	0.0 ± 0.0

**Table 2:** Minimum inhibitory concentration of *Lorus nobilis* methanolic extracts against *Escherichia coli* and *Staphylococcus aureus*.

		MIC (mg/mL)				MIC (mg/mL)	
No	Province	*E. coli*	*S. aureus*	No	Province	*E. coli*	*S. aureus*
1	Hatay1	12.5 ^d^	6.25 ^d^	48	Bartin1	12.5 ^d^	6.25 ^d^
2	Hatay2	12.5 ^d^	6.25 ^d^	49	Bartin2	6.25 ^e^	6.25 ^d^
3	Hatay3	12.5 ^d^	6.25 ^d^	50	Bartin3	12.5 ^d^	6.25 ^d^
4	K.maras	12.5 ^d^	3.125 ^e^	51	Zonguldak1	25 ^c^	6.25 ^d^
5	Mersin1	25 ^c^	6.25 ^d^	52	Zonguldak2	50 ^b^	12.5 ^c^
6	Mersin2	12.5 ^d^	12.5 ^c^	53	Zonguldak3	25 ^c^	6.25 ^d^
7	Mersin3	6.25 ^e^	3.125 ^e^	54	Zonguldak4	25 ^c^	6.25 ^d^
8	Mersin4	25 ^c^	3.125 ^e^	55	Zonguldak5	6.25 ^e^	3.125 ^e^
9	Antalya1	12.5 ^d^	12.5 ^c^	56	Duzce	6.25 ^e^	12.5 ^c^
10	Antalya2	12.5 ^d^	3.125 ^e^	57	Sakarya1	25 ^c^	12.5 ^c^
11	Antalya3	25 ^c^	3.125 ^e^	58	Sakarya2	12.5 ^d^	3.125 ^e^
12	Antalya4	25 ^c^	3.125 ^e^	59	Kocaeli1	50 ^b^	25 ^b^
13	Antalya5	25 ^c^	6.25 ^d^	60	Istanbul1	12.5 ^d^	3.125 ^e^
14	Antalya6	25 ^c^	6.25 ^d^	61	Istanbul2	6.25 ^e^	6.25 ^d^
15	Mugla1	6.25 ^e^	3.125 ^e^	62	Istanbul3	12.5 ^d^	6.25 ^d^
16	Mugla2	6.25 ^e^	3.125 ^e^	63	Kocaeli2	12.5 ^d^	3.125 ^e^
17	Mugla3	12.5 ^d^	6.25 ^d^	64	Kocaeli3	6.25 ^e^	6.25 ^d^
18	Mugla4	12.5 ^d^	12.5 ^c^	65	Yalova1	12.5 ^d^	3.125 ^e^
19	Aydin1	100 ^a^	6.25 ^d^	66	Yalova2	6.25 ^e^	3.125 ^e^
20	Izmir1	25 ^c^	3.125 ^e^	67	Bursa1	6.25 ^e^	6.25 ^d^
21	Izmir2	25 ^c^	3.125 ^e^	68	Bursa2	6.25 ^e^	6.25 ^d^
22	Giresun1	50 ^b^	6.25 ^d^	69	Balıkesir1	6.25 ^e^	12.5 ^c^
23	Giresun2	6.25 ^e^	3.125 ^e^	70	Balıkesir2	12.5 ^d^	6.25 ^d^
24	Giresun3	6.25 ^e^	3.125 ^e^	71	Izmir3	12.5 ^d^	6.25 ^d^
25	Trabzon1	6.25 ^e^	6.25 ^d^	72	Izmir4	6.25 ^e^	3.125 ^e^
26	Rize	50 ^b^	6.25 ^d^	73	Aydin2	12.5 ^d^	3.125 ^e^
27	Trabzon2	25 ^c^	3.125 ^e^	74	Aydin3	25 ^c^	25 ^b^
28	Trabzon3	25 ^c^	3.125 ^e^	75	Balıkesir1	50 ^b^	3.125 ^e^
29	Ordu1	25 ^c^	6.25 ^d^	76	Balıkesir2	12.5 ^d^	3.125 ^e^
30	Ordu2	25 ^c^	12.5 ^c^	77	Canakkale1	6.25 ^e^	6.25 ^d^
31	Samsun1	6.25 ^e^	12.5 ^c^	78	Canakkale2	25 ^c^	6.25 ^d^
32	Samsun2	12.5 ^d^	6.25 ^d^	79	Canakkale3	12.5 ^d^	12.5 ^c^
33	Samsun3	12.5 ^d^	3.125 ^e^	80	Canakkale4	12.5 ^d^	12.5 ^c^
34	Samsun4	6.25 ^e^	3.125 ^e^	81	Tekirdag1	25 ^c^	6.25 ^d^
35	Samsun5	25 ^c^	6.25 ^d^	82	Tekirdag2	12.5 ^d^	12.5
36	Samsun6	12.5 ^d^	6.25 ^d^	83	Tekirdag3	25 ^c^	6.25 ^d^
37	Sinop1	12.5 ^d^	12.5 ^c^	84	Tekirdag4	12.5 ^d^	6.25 ^d^
38	Sinop2	12.5 ^d^	25 ^b^	85	Tekirdag5	25 ^c^	3.125 ^e^
39	Sinop3	12.5 ^d^	12.5 ^c^	86	Istanbul4	12.5 ^d^	12.5 ^c^
40	Sinop4	25 ^c^	12.5 ^c^	87	Istanbul5	25 ^c^	25 ^b^
41	Sinop5	6.25 ^e^	12.5 ^c^	88	Istanbul6	6.25 ^e^	50 ^a^
42	Kastamonu1	12.5 ^d^	6.25 ^d^	89	Bursa3	50 ^b^	25 ^b^
43	Kastamon2	25 ^c^	6.25 ^d^	90	Yalova3	25 ^c^	12.5 ^c^
44	Kastamonu3	12.5 ^d^	3.125 ^e^	91	Bursa4	6.25 ^e^	6.25 ^d^
45	Kastamonu4	12.5 ^d^	6.25 ^d^	92	Izmir6	25 ^c^	6.25 ^d^
46	Kastamonu5	12.5 ^d^	3.125 ^e^				
47	Kastamonu6	6.25 ^e^	6.25 ^d^				

For each bacterial trait, mean values with a common letter are not significantly different at *p* ≤ 0.05.

**Table 3:** Evaluation of region-based diversity indices for Turkish laurel germplasm.

Regions	Na	Ne	I	He	uHe	P%
Mediterranean	1.648	1.431	0.380	0.253	0.262	74.82
Aegean	1.812	1.570	0.477	0.324	0.338	85.84
Black Sea	1.950	1.626	0.529	0.358	0.364	96.17
Marmara	1.912	1.589	0.500	0.338	0.344	93.19
All	1.830	1.554	0.471	0.318	0.327	87.50

Na: number of different alleles; Ne: number of effective alleles; I: Shannon’s index of information; He: expected heterozygosity; uHe: unbiased expected heterozygosity; P%: population percentage.

**Table 4:** Molecular analysis of variance (AMOVA) reveals genetic diversity in Turkish laurel germplasm.

Source	df	SS	MS	Est. Var.	%
Between populations	3	31,508.585	10,502.862	362.579	13%
Within populations	90	221,333.926	2459.266	2459.266	87%
Total	93	252,842.511		2821.845	100%

**Table 5:** Significant marker-trait associations identified by GWAS for antibacterial traits in 92 *Laurus nobilis* accessions.

Trait	Marker	*p*-Value	MarkerR^2^	Genetic Var (%)
EC-MEAN	26557159	*1.10* × *10*^−*4*^	0.18	9.4
EC-MEAN	26574788	*1.66* × *10*^−*4*^	0.20	9.4
SA-MEAN	26584578	*0.0018*	0.13	2.16 × 10^−4^
SA-MEAN	26565222	*0.00275*	0.11	2.16 × 10^−4^
BC-MEAN	26584774	*8.89* × *10*^−*5*^	0.19	12.5
EC-MIC-Mean	26584078	*3.80* × *10*^−*4*^	0.15	54.6
EC-MIC-Mean	26569339	*5.13* × *10*^−*4*^	0.18	54.6
EC-MIC-Mean	26583551	*5.24* × *10*^−*4*^	0.16	54.6
SA-MIC-Mean	26567210	*1.13* × *10*^−*4*^	0.21	18.8
SA-MIC-Mean	26563218	*1.30* × *10*^−*4*^	0.18	18.8
SA-MIC-Mean	26586695	*1.95* × *10*^−*4*^	0.23	18.8

**Table 6:** Performance metrics for machine learning models predicting the antibacterial activity of *Laurus nobilis* extracts.

**RF**					
	**R^2^**	**RMSE**	**MAE**	**MLSE**	**MedAE**
**MIC**	0.968	2.180	0.968	0.030	0.065
**Disc diffusion**	0.984	0.934	0.554	0.004	0.000
**LightGBM**					
	**R^2^**	**RMSE**	**MAE**	**MLSE**	**MedAE**
**MIC**	0.988	1.315	0.304	0.008	0.117
**Disc diffusion**	0.695	4.045	3.273	0.414	2.740
**XGBoost**					
	**R^2^**	**RMSE**	**MAE**	**MLSE**	**MedAE**
**MIC**	0.999	0.434	0.380	0.003	0.394
**Disc diffusion**	0.781	3.429	2.576	0.189	1.872

R^2^: coefficient of determination; RMSE: root mean squared error; MAE: mean absolute error; MSLE: mean squared logarithmic error; MedAE: median absolute error; RF: random forest; MIC: minimum inhibitory concentration.

## Data Availability

The original contributions presented in this study are included in the article/Supplementary Materials. Further inquiries can be directed to the corresponding author.

## Supplementary Materials

The following supporting information can be downloaded at: https://www.mdpi.com/article/10.3390/plants15131997/s1, Table S1: Antibacterial activity of laurel methanolic extracts against three bacterial strains measured by disc diffusion method; Table S2: Collection sites and passport information of the plant materials; Table S3: Plant material collected location with all possible features.

## References

[1] Stefanova G., Girova T., Gochev V., Stoyanova M., Petkova Z., Stoyanova A., Zheljazkov V.D. (2020). Comparative study on the chemical composition of laurel (*Laurus nobilis* L.) leaves from Greece and Georgia and the antibacterial activity of their essential oil. Heliyon.

[2] Shahrajabian M.H., Sun W. (2023). Survey on medicinal plants and herbs in traditional Iranian medicine with anti-oxidant, anti-viral, anti-microbial, and anti-inflammation properties. Lett. Drug Des. Discov..

[3] Paparella A., Nawade B., Shaltiel-Harpaz L., Ibdah M. (2022). A review of the botany, volatile composition, biochemical and molecular aspects, and traditional uses of *Laurus nobilis*. Plants.

[4] Abou-Khalil R., Al Hayek D., Al Hakim M., Andary J. (2026). Lebanese Bay Leaves (*Laurus nobilis*): A Unique Chemotypic and Pharmacological Profile with Culinary and Medicinal Potential. J. Food Compos. Anal..

[5] Akinboye A.O., Adeyemo R.O., Karzis J., Petzer I.M., McGaw L.J. (2024). Susceptibility patterns of Escherichia coli and streptococcal isolates from bovine mastitis cases to antibiotics and selected South African plant extracts with known antibacterial activities. S. Afr. J. Bot..

[6] Divya M., Shanti G., Amalraj S., Amiri-Ardekani E., Gurav S., Ayyanar M. (2023). Evaluation of in vitro enzyme inhibitory, anti-inflammatory, antioxidant, and antibacterial activities of *Oldenlandia corymbosa* L. and *Oldenlandia umbellata* L. whole plant extracts. Pharmacol. Res. Mod. Chin. Med..

[7] Kavitha H.U., Satish S. (2011). Eco-friendly management of plant pathogens by some medicinal plant extracts. J. Agric. Technol..

[8] Karimou R., Salami H., Agossou E., Boya B., Assouma F., Bio Bouko B.O.M., Attakpa E., Baba-Moussa L., Sina H. (2024). Assessment of anti-microbial and anti-biofilm activities of lemongrass and bay leaf extracts on microorganisms from fermented cereal-based porridges in Northern Benin. Sci. Afr..

[9] Hossain T.J. (2024). Methods for screening and evaluation of anti-microbial activity: A review of protocols, advantages, and limitations. Eur. J. Microbiol. Immunol..

[10] Sun L., Lai M., Ghouri F., Nawaz M.A., Ali F., Baloch F.S., Nadeem M.A., Aasim M., Shahid M.Q. (2024). Modern plant breeding techniques in crop improvement and genetic diversity: From molecular markers and gene editing to artificial intelligence—A critical review. Plants.

[11] LeCun Y., Bengio Y., Hinton G. (2015). Deep learning. Nature.

[12] McKinney B.A., Reif D.M., Ritchie M.D., Moore J.H. (2006). Machine learning for detecting gene-gene interactions: A review. Appl. Bioinform..

[13] Stokes J.M., Yang K., Swanson K., Jin W., Cubillos-Ruiz A., Donghia N.M., MacNair C.R., French S., Carfrae L.A., Bloom-Ackermann Z. (2020). A deep learning approach to antibiotic discovery. Cell.

[14] Yu J., Buckler E.S. (2006). Genetic association mapping and genome organization of maize. Curr. Opin. Biotechnol..

[15] Zila C.T., Ogut F., Romay M.C., Gardner C.A., Buckler E.S., Holland J.B. (2014). Genome-wide association study of Fusarium ear rot disease in the USA maize inbred line collection. BMC Plant Biol..

[16] Chen G., Wang X., Hao J., Yan J., Ding J. (2015). Genome-wide association implicates candidate genes conferring resistance to maize rough dwarf disease in maize. PLoS ONE.

[17] Kaul S., Choudhary M., Gupta S., Dhar M.K. (2021). Engineering host microbiome for crop improvement and sustainable agriculture. Front. Microbiol..

[18] Maciel-Guerra A., Baker M., Hu Y., Wang W., Zhang X., Rong J., Liguori K., Guo Z., Liu X., Dong P. (2023). Dissecting microbial communities and resistomes for interconnected humans, soil, and livestock. ISME J..

[19] Rampone S., Pagliarulo C., Marena C., Orsillo A., Iannaccone M., Trionfo C., Pellegrini B., Nacca M., Volpe M.G. (2021). In silico analysis of the anti-microbial activity of phytochemicals: Towards a technological breakthrough. Comput. Methods Programs Biomed..

[20] Sun Z., Hong W., Xue C., Dong N. (2024). A comprehensive review of antibiotic resistance gene contamination in agriculture: Challenges and AI-driven solutions. Sci. Total Environ..

[21] Bombrun M., Dash J.P., Pont D., Watt M.S., Pearse G.D., Dungey H.S. (2020). Forest-scale phenotyping: Productivity characterisation through machine learning. Front. Plant Sci..

[22] Ratner B. (2017). Statistical and Machine-Learning Data Mining: Techniques for Better Predictive Modeling and Analysis of Big Data.

[23] Anahtar M.N., Yang J.H., Kanjilal S. (2021). Applications of machine learning to the problem of antimicrobial resistance: An emerging model for translational research. J. Clin. Microbiol..

[24] Abebe A., Teklay Hilawea K., Mekonnen A., Tamiru Tigineh G., Sitotaw B., Liyew M., Wubieneh T.A. (2022). Assessment on antioxidant activity of the aqueous leaf extracts of Combretum microphyllum and the effect of Co(II)-leaf extract complex on antibacterial activity of leaf extracts of the plant material. Sci. Afr..

[25] Ibrahim N., Kebede A. (2020). In vitro antibacterial activities of methanol and aqueous leave extracts of selected medicinal plants against human pathogenic bacteria. Saudi J. Biol. Sci..

[26] Ramos C., Teixeira B., Batista I., Matos O., Serrano C., Neng N.R., Nogueira J.M.F., Nunes M.L., Marques A. (2012). Antioxidant and antibacterial activity of essential oil and extracts of bay laurel *Laurus nobilis* Linnaeus (Lauraceae) from Portugal. Nat. Prod. Res..

[27] Ozcan B., Esen M., Sangun M.K., Coleri A., Caliskan M. (2010). Effective antibacterial and antioxidant properties of methanolic extract of *Laurus nobilis* seed oil. J. Environ. Biol..

[28] Al-Bayati F.A. (2008). Synergistic antibacterial activity between Thymus vulgaris and Pimpinella anisum essential oils and methanol extracts. J. Ethnopharmacol..

[29] Liu Y., McKeever L.C., Malik N. (2017). Assessment of the anti-microbial activity of olive leaf extract against foodborne bacterial pathogens. Front. Microbiol..

[30] Coccimiglio J., Alipour M., Jiang Z., Gottardo C., Suntres Z. (2016). Antioxidant, antibacterial, and cytotoxic activities of the ethanolic Origanum vulgare extract and its major constituents. Biomed. Res. Int..

[31] Singh A.A., Naaz Z.T., Rakaseta E., Perera M., Singh V., Cheung W., Chand A., Waqainabete L., Sharma N., Mudaliar V. (2023). Anti-microbial activity of selected plant extracts against common food borne pathogenic bacteria. Food Humanit..

[32] Xedzro C., Tano-Debrah K., Nakano H. (2022). Antibacterial efficacies and time-kill kinetics of indigenous Ghanaian spice extracts against Listeria monocytogenes and some other food-borne pathogenic bacteria. Microbiol. Res..

[33] Fantasma F., Samukha V., Aliberti M., Colarusso E., Chini M.G., Saviano G., Bifulco G., Lauro G., Montoro P., Skhirtladze A. (2024). Essential oils of *Laurus nobilis* L.: From chemical analysis to in silico investigation of anti-inflammatory activity by soluble epoxide hydrolase (sEH) inhibition. Foods.

[34] Bojović D., Šoškić M., Žugić A., Milenković M.T., Ljumović I., Tadić V.M. (2025). Chemical analysis and anti-microbial potential assessment of wild laurel from the National Park Skadar Lake, Montenegro. Appl. Sci..

[35] Esertaş Ü.Z., Cora M. (2024). Biological activities of Elaeagnus umbellata methanol extract. Kahramanmaraş Sütçü İmam Univ. J. Agric. Nat..

[36] Daştan T., Kaya Ş., Öz M., Daştan S.D. (2025). Determination of the bioavailability of *Arum maculatum* L. plant extracts in Caenorhabditis elegans culture. Turk. J. Agric. Food Sci. Technol..

[37] Jaradat N., Hawash M., Qaoud M.T., Al-Maharik N., Qadi M., Hussein F., Issa L., Hamdan Z., Shawahna R., Al-Lahham S. (2024). Biological, phytochemical and molecular docking characteristics of *Laurus nobilis* L. fresh leaves essential oil from Palestine. BMC Complement. Med. Ther..

[38] Karık Ü., Nadeem M.A., Habyarimana E., Ercişli S., Yildiz M., Yılmaz A., Yang S.H., Chung G., Baloch F.S. (2019). Exploring the genetic diversity and population structure of Turkish laurel germplasm by the iPBS-retrotransposon marker system. Agronomy.

[39] Yilmaz A., Ciftci V. (2021). Genetic relationships and diversity analysis in Turkish laurel (*Laurus nobilis* L.) germplasm using ISSR and SCoT markers. Mol. Biol. Rep..

[40] Rego R., Vieira Â.F., Silva L., Elias R.B., Silva C., Resendes R., Moura M. (2024). Microsatellites reveal high levels of genetic admixture in the natural populations of Laurus azorica, Lauraceae. Plant Syst. Evol..

[41] Megha, Singh N., Sharma M., Langyan S., Kumar Singh N. (2024). Genome wide association study of antioxidant activity in pigeonpea germplasm. Discov. Food.

[42] Nadeem M.A., Gündoğdu M., Ercişli S., Karaköy T., Saracoğlu O., Habyarimana E., Nawaz M.A., Alsaleh A., Chung G., Baloch F.S. (2019). Uncovering phenotypic diversity and DArTseq marker loci associated with antioxidant activity in common bean. Genes.

[43] Habyarimana E., Dall’Agata M., De Franceschi P., Baloch F.S. (2019). Genome-wide association mapping of total antioxidant capacity, phenols, tannins, and flavonoids in a panel of *Sorghum bicolor* and *S. bicolor* × *S. halepense* populations using multi-locus models. PLoS ONE.

[44] Yadav C.B., Tokas J., Yadav D., Winters A., Singh R.B., Yadav R., Bhardwaj R., Srivastava R.K. (2021). Identifying anti-oxidant biosynthesis genes in pearl millet [*Pennisetum glaucum* (L.) R. Br.] using genome-wide association analysis. Front. Plant Sci..

[45] Ro N., Haile M., Ko H.C., Cho G.T., Lee J., Kim B., Rhee J.H., Ko J.M. (2023). Genome-wide association study of phenolic content and antioxidant properties in eggplant germplasm. Genes.

[46] Marcotuli I., Vurro F., Mores A., Pasqualone A., Colasuonno P., Cabas-Lühmann P., Gadaleta A. (2025). Genetic study of total phenolic content and antioxidant activity traits in tetraploid wheat via genome-wide association mapping. Antioxidants.

[47] Nogueira A.F., Moda-Cirino V., dos Santos Neto J., Delfini J., Fagundes D.F.V., Gepts P., Gonçalves L.S.A. (2025). Association mapping for total phenols, total flavonoids and antioxidant activity in a Mesoamerican bean diversity panel. Front. Agron..

[48] Takemoto K., Ebine K., Askani J.C., Krüger F., Gonzalez Z.A., Ito E., Goh T., Schumacher K., Nakano A., Ueda T. (2018). Distinct sets of tethering complexes, SNARE complexes, and Rab GTPases mediate membrane fusion at the vacuole in Arabidopsis. Proc. Natl. Acad. Sci. USA.

[49] Barbez E., Kubeš M., Rolčík J., Béziat C., Pěnčík A., Wang B., Rosquete M.R., Zhu J., Dobrev P.I., Lee Y. (2012). A novel putative auxin carrier family regulates intracellular auxin homeostasis in plants. Nature.

[50] Yu G.Y., Lee G.W., Hung Y.T., Li S.C., Ma Y.P., Chen Z.W., Hsuan S.L. (2025). AI-driven identification and analysis of inhibition zones in disk diffusion tests with the hue contrast method. Microchem. J..

[51] Liu B., Liu Y., Xu S., Wu Q., Wu D., Zhan L., Chen M., Huang T., Guan J., Zhou T. (2025). EasyMultiProfiler: An efficient multi-omics data integration and analysis workflow for microbiome research. Sci. China Life Sci..

[52] Van Klompenburg T., Kassahun A., Catal C. (2020). Crop yield prediction using machine learning: A systematic literature review. Comput. Electron. Agric..

[53] Raschka S. (2018). Model evaluation, model selection, and algorithm selection in machine learning. arXiv.

[54] Liakos K.G., Busato P., Moshou D., Pearson S., Bochtis D. (2018). Machine learning in agriculture: A review. Sensors.

[55] Islam N., Rashid M.M., Wibowo S., Xu C.Y., Morshed A., Wasimi S.A., Moore S., Rahman S.M.E. (2021). Early weed detection using image processing and machine learning techniques in an Australian chilli farm. Agriculture.

[56] Guo F., Mo H., Wu J., Pan L., Zhou H., Zhang Z., Chen B., Li X., Zhou S., Zhang Z. (2024). A hybrid stacking model for enhanced short-term load forecasting. Electronics.

[57] Zhang L., Li R., Li Z., Meng Y., Liang J., Fu L., Zhang X., Fang S., Li F., Zhang H. (2021). A quadratic traversal algorithm of shortest weeding path planning for agricultural mobile robots in cornfield. J. Robot..

[58] Bowers J.S., Malhotra G., Dujmović M., Montero M.L., Tsvetkov C., Biscione V., Puebla G., Adolfi F., Hummel J.E., Heaton R.F. (2023). Deep problems with neural network models of human vision. Behav. Brain Sci..

[59] Ngamsurach P., Praipipat P. (2022). Antibacterial activities against Staphylococcus aureus and Escherichia coli of extracted Piper betle leaf materials by disc diffusion assay and batch experiments. RSC Adv..

[60] Güler Ş., Torul D., Kurt-Bayrakdar S., Tayyarcan E.K., Çamsarı Ç., Boyacı İ.H. (2023). Evaluation of antibacterial efficacy of Lawsonia inermis Linn (henna) on periodontal pathogens using agar well diffusion and broth microdilution methods: An in-vitro study. BioMedicine.

[61] El-Shamy H.A., Hashish M.H., Abaza A.F., Dorra N.H. (2013). Antibacterial activity of medicinal herb and spice extracts. Int. J. Food Nutr. Public Health.

[62] Baloch F.S., Lee S.M., Mansoor S., Morales A., Karunathilake E.M.B.M., Nadeem M.A., Habyarimana E., Nawaz M.A., Chung G. (2025). GBS-derived SNP and SilicoDArT markers reveals the genetic variation and population structure of Korean buckwheat (*Fagopyrum esculentum*) an underutilised crop. BMC Plant Biol..

[63] Doyle J.J. (1990). Isolation of plant DNA from fresh tissue. Focus.

[64] Maniatis T., Fritsch E.F., Sambrook J. (1982). Molecular Cloning: A Laboratory Manual.

[65] Kilian A., Wenzl P., Huttner E., Carling J., Xia L., Blois H., Caig V., Heller-Uszynska K., Jaccoud D., Hopper C., Pompanon F., Bonin A. (2012). Diversity arrays technology: A generic genome profiling technology on open platforms. Data Production and Analysis in Population Genomics: Methods and Protocols.

[66] Li X., Yang Y., Henry R.J., Rossetto M., Wang Y., Chen S. (2015). Plant DNA barcoding: From gene to genome. Biol. Rev..

[67] Pritchard J.K., Stephens M., Donnelly P. (2000). Inference of population structure using multilocus genotype data. Genetics.

[68] Evanno G., Regnaut S., Goudet J. (2005). Detecting the number of clusters of individuals using the software STRUCTURE: A simulation study. Mol. Ecol..

[69] Bradbury P.J., Zhang Z., Kroon D.E., Casstevens T.M., Ramdoss Y., Buckler E.S. (2007). Tassel: Software for association mapping of complex traits in diverse samples. Bioinformatics.

[70] Lamesch P., Berardini T.Z., Li D., Swarbreck D., Wilks C., Sasidharan R., Muller R., Dreher K., Alexander D.L., Garcia-Hernandez M. (2012). The Arabidopsis Information Resource (TAIR): Improved gene annotation and new tools. Nucleic Acids Res..

[71] Szklarczyk D., Gable A.L., Lyon D., Junge A., Wyder S., Huerta-Cepas J., Simonovic M., Doncheva N.T., Morris J.H., Bork P. (2019). STRING v11: Protein–protein association networks with increased coverage, supporting functional discovery in genome-wide experimental datasets. Nucleic Acids Res..

[72] von Mering C., Jensen L.J., Snel B., Hooper S.D., Krupp M., Foglierini M., Jouffre N., Huynen M.A., Bork P. (2005). STRING: Known and predicted protein–protein associations, integrated and transferred across organisms. Nucleic Acids Res..

[73] Thomas P.D., Ebert D., Muruganujan A., Mushayahama T., Albou L.P., Mi H. (2022). PANTHER: Making genome-scale phylogenetics accessible to all. Protein Sci..

[74] Breiman L. (2001). Random forests. Mach. Learn..

[75] Ke G., Meng Q., Finley T., Wang T., Chen W., Ma W., Ye Q., Liu T.Y. (2017). Lightgbm: A highly efficient gradient boosting decision tree. Adv. Neural Inf. Process. Syst..

[76] Chen T., Guestrin C. Xgboost: A scalable tree boosting system. Proceedings of the 22nd ACM SIGKDD International Conference on Knowledge Discovery and Data Mining.

[77] Hastie T., Tibshirani R., Friedman J. (2009). The Elements of Statistical Learning.

[78] Altaf M.T., Aasim M., Ali S.A., Say A., Soomro S.R., Soomro S.N., Demir M., Liaqat W., Aadil F., Horoz S. (2026). Optimizing biomass production and antioxidant dynamics in *Ceratophyllum demersum* L. using multi-walled carbon nanotubes and machine learning models. BMC Plant Biol..

[79] Şavşatlı Y., Aasim M., Katırcı R., Talaz O., Altaf M.T. (2026). Machine learning and mathematical modeling for comparative analysis of green-synthesized ZnO nanoparticles as seed nano-priming agents for linseed. Front. Plant Sci..

