# Supplementary material for: Genetic Diversity, Population Structure, Integration of Genome-Wide Association Studies and Machine Learning for Antibacterial Trait Analysis in the Mediterranean Spice Laurel (Laurus nobilis)
Source: Plants (Basel). 2026 Jun 27;15(13):1997. doi: 10.3390/plants15131997 (PMC13363779; doi:10.3390/plants15131997)
Supplement: Supplementary file 1 [file plants-15-01997-s001.zip › plants-4309606-supplementary.pdf]

Supplementary table S1. Antibacterial activity of laurel methanolic extracts against three bacterial strains measured by disk diffusion method.

Acessions		Mean of inhibition zone ± SD (mm)		
**No**	**Name***	***E. coli***	***S. aureus***	***B. cereus***
1	Hatay1	23.0 ± 0.0 b	8.0 ± 0.0 st	16.0 ± 0.0 b
2	Hatay2	19.5 ± 0.5 gh	14.5 ± 0.5 mn	13.0 ± 1.0 d
3	Hatay3	20.0 ± 0.0 fg	15.0 ± 0.0 m	12.0 ± 0.0 e
4	Kahramanmaras	17.5 ± 0.5 jk	25.0 ± 1.0 a-c	0.0 ± 0.0 m
5	Mersin1	14.5 ± 0.5 l-n	18.0 ± 0.0 j	12.0 ± 0.0 e
6	Mersin2	14.5 ± 0.5 l-n	15.5 ± 0.5 lm	13.0 ± 1.0 d
7	Mersin3	14.0 ± 0.0 m-o	15.0 ± 0.0 m	11.0 ± 1.0 f
8	Mersin4	18.5 ± 0.5 h-j	10.5 ± 1.5 p-r	10.0 ± 0.0 g
9	Antalya1	12.0 ± 0.0 q-s	24.0 ± 1.0 b-d	16.0 ± 0.0 b
10	Antalya2	15.0 ± 0.0 lm	18.5 ± 0.5 ij	8.0 ± 0.0 h
11	Antalya3	17.5 ± 0.5 jk	18.0 ± 0.0 j	0.0 ± 0.0 m
12	Antalya4	10.0 ± 0.0 uv	20.5 ± 0.5 gh	12.0 ± 0.0 e
13	Antalya5	14.5 ± 0.5 l-n	15.0 ± 1.0 m	10.0 ± 0.0 g
14	Antalya6	14.0 ± 0.0 m-o	18.0 ± 0.0 j	0.0 ± 0.0 m
15	Mugla1	22.5 ± 0.5 b-d	*7.5 ± 0.5* t	0.0 ± 0.0 m
16	Mugla2	21.0 ± 1.0 ef	13.0 ± 1.0 no	6.0 ± 0.0 j
17	Mugla3	18.0 ± 1.0 i-k	15.5 ± 0.5 lm	0.0 ± 0.0 m
18	Mugla4	8.5 ± 0.5 wx	9.0 ± 1.0 r-t	0.0 ± 0.0 m
19	Aydin1	21.5 ± 0.5 de	24.5 ± 0.5 a-d	4.5 ± 0.5 k
20	Izmir1	12.5 ± 0.5 p-r	18.5 ± 0.5 ij	11.0 ± 1.0 f
21	Izmir2	15.5 ± 0.5 l	18.0 ± 0.0 j	8.5 ± 0.5 h
22	Giresun1	10.0 ± 0.0 uv	20.5 ± 0.5 gh	7.0 ± 0.0 i
23	Giresun2	15.5 ± 0.5 l	22.0 ± 0.0 e-g	12.0 ± 0.0 e
24	Giresun3	14.0 ± 0.0 m-o	18.5 ± 0.5 ij	16.0 ± 0.0 b
25	Trabzon1	12.5 ± 0.5 p-r	18.0 ± 0.0 j	0.0 ± 0.0 m
26	Rize	18.0 ± 0.0 i-k	14.5 ± 0.5 mn	0.0 ± 0.0 m
27	Trabzon2	21.5 ± 0.5 de	21.5 ± 0.5 f-h	0.0 ± 0.0 m
28	Trabzon3	20.0 ± 0.0 fg	18.0 ± 0.0 j	0.0 ± 0.0 m
29	Ordu1	15.0 ± 0.0 lm	15.0 ± 0.0 m	0.0 ± 0.0 m
30	Ordu2	14.0 ± 0.0 m-o	15.5 ± 0.5 lm	0.0 ± 0.0 m
31	Samsun1	7.5 ± 0.5 xy	23.0 ± 1.0 d-f	8.0 ± 0.0 h
32	Samsun2	14.0 ± 0.0 m-o	20.5 ± 0.5 gh	5.0 ± 1.0 k
33	Samsun3	15.0 ± 0.0 lm	24.5 ± 0.5 a-d	0.0 ± 0.0 m
34	Samsun4	14.5 ± 0.5 l-n	17.5 ± 0.5 jk	0.0 ± 0.0 m
35	Samsun5	11.5 ± 0.5 r-t	18.0 ± 0.0 j	0.0 ± 0.0 m
36	Samsun6	14.5 ± 0.5 l-n	23.5 ± 0.5 c-e	4.5 ± 0.5 k
37	Sinop1	*7.0 ± 0.0 y*	14.5 ± 0.5 mn	0.0 ± 0.0 m
38	Sinop2	11.5 ± 0.5 r-t	8.0 ± 0.0 st	0.0 ± 0.0 m
39	Sinop3	8.5 ± 0.5 wx	15.0 ± 0.0 m	0.0 ± 0.0 m
40	Sinop4	9.5 ± 0.5 vw	15.0 ± 0.0 m	0.0 ± 0.0 m
41	Sinop5	8.5 ± 0.5 wx	14.5 ± 0.5 mn	0.0 ± 0.0 m
42	Kastamonu1	12.0 ± 0.0 q-s	17.5 ± 0.5 jk	0.0 ± 0.0 m
43	Kastamonu2	14.0 ± 0.0 m-o	18.5 ± 1.5 ij	0.0 ± 0.0 m

44	Kastamonu3	12.0 ± 0.0 q-s	16.0 ±1.0 k-m	0.0 ± 0.0 m
45	Kastamonu4	12.0 ± 0.0 q-s	15.0 ± 0.0 m	0.0 ± 0.0 m
46	Kastamonu5	14.0 ± 0.0 m-o	15.0 ± 0.0 m	0.0 ± 0.0 m
47	Kastamonu6	11.0 ± 1.0 s-u	15.5 ± 0.5 lm	0.0 ± 0.0 m
48	Bartin1	18.0 ± 0.0 i-k	12.0 ± 0.0 op	2.0 ± 0.0 l
49	Bartin2	17.0 ± 0.0 k	14.5 ± 0.5 mn	0.0 ± 0.0 m
50	Bartin3	14.5 ± 0.5 l-n	14.5 ± 0.5 mn	2.0 ± 0.0 l
51	Zonguldak1	12.5 ± 0.5 p-r	23.0 ± 1.0 d-f	0.0 ± 0.0 m
52	Zonguldak2	12.0 ± 0.0 q-s	21.0 ± 1.0 gh	0.0 ± 0.0 m
53	Zonguldak3	10.0 ± 0.0 uv	18.0 ± 0.0 j	0.0 ± 0.0 m
54	Zonguldak4	13.5 ± 0.5 n-p	25.5 ± 0.5 ab	0.0 ± 0.0 m
55	Zonguldak5	12.0 ± 0.0 q-s	18.5 ± 0.5 ij	0.0 ± 0.0 m
56	Duzce	19.5 ± 0.5 gh	11.5 ± 0 .5 op	0.0 ± 0.0 m
57	Sakarya1	17.0 ± 0.0 k	12.0 ± 0.0 op	8.0 ± 0.0 h
58	Sakarya2	18.0 ± 0.0 i-k	14.5 ± 0.5 mn	7.0 ± 0.0 i
59	Kocaeli1	14.0 ± 0.0 m-o	24.0 ± 0.0 b-d	12.0 ± 0.0 e
60	Istanbul1	8.0 ± 0.0 xy	11.5 ± 0.5 op	0.0 ± 0.0 m
61	Istanbul2	12.0 ± 0.0 q-s	10.5 ± 0.5 p-r	0.0 ± 0.0 m
62	Istanbul3	13.0 ± 0.0 o-q	12.0 ± 0.0 op	0.0 ± 0.0 m
63	Kocaeli2	18.0 ± 0.0 i-k	12.0 ± 0.0 op	0.0 ± 0.0 m
64	Kocaeli3	19.0 ± 0.0 g-i	13.0 ± 1.0 no	10.0 ± 0.0 g
65	Yalova1	18.0 ± 0.0 i-k	12.0 ± 0.0 op	0.0 ± 0.0 m
66	Yalova2	17.5 ± 0.0jk	13.0 ± 1.0 no	0.0 ± 0.0 m
67	Bursa1	14.5 ± 0.5l -n	11.0 ± 1.0 pq	0.0 ± 0.0 m
68	Bursa2	14.0 ± 0.0 m-o	14.5 ± 0.5 mn	0.0 ± 0.0 m
69	Balıkesir1	12.0 ± 0.0 q-s	17.5 ± 0.5 jk	**17.0 ± 1.0 a**
70	Balıkesir2	10.0 ± 0.0 uv	18.0 ± 0.0 j	12.0 ± 0.0 e
71	Izmir3	18.5 ± 0.5 h-j	23.0 ± 1.0 d-f	16.0 ±0.0 b
72	Izmir4	22.0 ± 0.0 c-e	**26.0 ± 1.0 a**	14.5 ± 0.5 c
73	Aydin2	23.5 ± 0.5 ab	**26.0 ± 2.0 a**	12.0 ± 0.0 e
74	Aydin3	22.0 ± 0.0 c-e	21.5 ± 0.5 f-h	11.0 ± 1.0 f
75	Balikesir1	15.0 ± 0.0 lm	21.0 ± 1.0 gh	14.0 ± 0.0 c
76	Balikesir2	15.0 ± 0.0 lm	20.0 ± 0.0 hi	10.0 ± 0.0 g
77	Canakkale1	20.0 ± 0.0 fg	20.0 ± 0.0 hi	8.0 ± 0.0 h
78	Canakkale2	**24.5 ± 0.5 a**	24.5 ± 0.5 a-d	6.0 ± 0.0 j
79	Canakkale3	21.0 ± 1.0 ef	17.0 ± 1.0 j-l	8.0 ± 0.0 h
80	Canakkale4	20.0 ± 0.0 fg	18.0 ± 0.0 j	6.0 ± 0.0 j
81	Tekirdag1	8.0 ± 0.0 xy	20.5 ± 0.5 gh	0.0 ± 0.0 m
82	Tekirdag2	10.0 ± 0.0 uv	20.0 ± 0.0 hi	0.0 ± 0.0 m
83	Tekirdag3	10.0 ± 0.0 uv	13.0 ± 1.0 no	0.0 ± 0.0 m
84	Tekirdag4	12.0 ± 0.0 q-s	23.0 ± 1.0 d-f	0.0 ± 0.0 m
85	Tekirdag5	14.5 ± 0.5 l-n	20.0 ± 0.0 hi	0.0 ± 0.0 m
86	Istanbul4	18.0 ± 0.0 i-k	9.5 ± 0.5 q-s	0.0 ± 0.0 m
87	Istanbul5	17.5 ± 0.5 jk	13.0 ± 1.0 no	0.0 ± 0.0 m
88	Istanbul6	15.5 ± 0.5 l	9.0 ± 1.0 r-t	0.0 ± 0.0 m
89	Bursa3	10.0 ± 1.0 uv	20.5 ± 0.5 gh	0.0 ± 0.0 m
90	Yalova3	10.0 ± 0.0 uv	18.0 ± 0.0 j	0.0 ± 0.0 m

91	Bursa4	10.5 ± 0.5 t-v	14.5 ± 0.5 mn	0.0 ± 0.0 m
92	Izmir6	11.5 ± 0.5 r-t	8.5 ± 0.5 st	12.0 ± 0.0 e
	**Mean ± SD**	**14.8 ± 4.2**	**16.9 ± 4.6**	**4.4 ± 5.6**
	**Range**	**7.0 – 24.5**	**7.5 – 26.0**	**0.0 – 17.0**

[1]Accession names denote their geographical origin (province) followed by a number indicating the individual plant introduction to the germplasm collection and the exact collection site (i.e., the district and location), as described in Table S2. Values are mean ± standard deviation from biological replicates. Mean values in bold indicate the strongest inhibition for a given bacterial strain, and values in italics indicate the lowest inhibitory effect. For each bacterial strains, mean values with a common letter are not significantly different at $p \leq 0.05$. E. coli, *Escherichia coli*; S. aureus, *Staphylococcus aureus*

Supplementary table S2. Membership coefficient (Q-matrix) of laurel genotypes resulting from structure analysis.

Genotype	Cluster1	Cluster2
Hatay2	1	0
Kahramanmar	1	0
Mersin1	1	0
Mersin3	1	0
Antalya1	1	0
Mersin4	0.999	0.001
Adana	0.979	0.021
Hatay1	0.975	0.025
Hatay3	0.928	0.072
Trabzon1	0.883	0.117
Antalya4	0.876	0.124
Trabzon2	0.865	0.135
Antalya5	0.854	0.146
Antalya2	0.849	0.151
Trabzon3	0.841	0.159
Ordu2	0.83	0.17
Mugla1	0.822	0.178
Ordu1	0.791	0.209
Antalya6	0.786	0.214
Giresun3	0.771	0.229
Samsun1	0.768	0.232
Rize	0.762	0.238
Samsun2	0.72	0.28
Antalya3	0.698	0.302
Giresun2	0.689	0.311
Samsun3	0.656	0.344
Yalova2	0.648	0.352
Sinop1	0.63	0.37

Samsun6	0.617	0.383
Samsun4	0.594	0.406
Samsun5	0.589	0.411
Kastamonu1	0.514	0.486
Mugla2	0.51	0.49
Sinop4	0.475	0.525
Sinop2	0.467	0.533
Sinop3	0.454	0.546
Kastamonu4	0.399	0.601
Kastamonu3	0.387	0.613
Kastamon2	0.377	0.623
Sinop5	0.352	0.648
Istanbul1	0.249	0.751
Kastamonu6	0.161	0.839
Zonguldak4	0.158	0.842
Zonguldak2	0.133	0.867
Aydin1	0.131	0.869
Sakarya1	0.123	0.877
Duzce	0.116	0.884
Kastamonu5	0.114	0.886
Bartin1	0.113	0.887
Bartin3	0.109	0.891
Mugla4	0.103	0.897
Kocaeli1	0.102	0.898
Mugla3	0.097	0.903
Kocaeli2	0.093	0.907
Zonguldak5	0.085	0.915
Mersin2	0.082	0.918
Zonguldak3	0.08	0.92
Bartin2	0.076	0.924
Yalova1	0.073	0.927
Zonguldak1	0.069	0.931
Bursa3	0.062	0.938
Izmir3	0.06	0.94
Izmir1	0.058	0.942
Balikesir1	0.042	0.958
Tekirdag3	0.041	0.959
Balikesir2	0.037	0.963
Izmir6	0.018	0.982
Balikesir1	0.017	0.983
Aydin3	0.017	0.983
Sakarya2	0.016	0.984
Bursa4	0.012	0.988

Canakkale1	0.011	0.989
Balikesir2	0.005	0.995
Izmir4	0.004	0.996
Istanbul2	0.002	0.998
Bursa1	0.002	0.998
Izmir5	0.002	0.998
Canakkale4	0.002	0.998
Tekirdag1	0.002	0.998
Istanbul4	0.002	0.998
Izmir2	0.001	0.999
Giresun1	0.001	0.999
Istanbul3	0.001	0.999
Bursa2	0.001	0.999
Aydin2	0.001	0.999
Canakkale2	0.001	0.999
Canakkale3	0.001	0.999
Tekirdag5	0.001	0.999
Istanbul5	0.001	0.999
Kocaeli3	0	1
Tekirdag2	0	1
Tekirdag4	0	1
Istanbul6	0	1
Yalova3	0	1

Supplementary Table S3. Plant material collected location with all possible features

No	Province	District	Location	Altitude (m)	No	Province	District	Location	Altitude (m)
1	Hatay	Samandagi	Yogunoluk	317	48	Bartin	Kurucasile	Kapisuyu	147
2	Hatay	Yayladagi	Yesiltepe	696	49	Bartin	Amasra	Kalesah	145
3	Hatay	Antakya	Harbiye	188	50	Bartin	Merkez	Karasu	23
4	K.maras	Andirin	Gokceli	971	51	Zonguldak	Merkez	Gokgol	164
5	Mersin	Toroslar	Musali	570	52	Zonguldak	Kilimli	Camlik	88
6	Mersin	Erdemli	Esenpinari	783	53	Zonguldak	Merkez	Kozlu	46
7	Mersin	Silifke	Demircili	411	54	Zonguldak	Alapli	Merkez	20
8	Mersin	Anamur	Korucuk	116	55	Zonguldak	Eregli	Guluc	9
9	Antalya	Gazipasa	Demirtas	281	56	Duzce	Akcakoca	Merkez	10

10	Antalya	Manavgat	Sarilar	2	57	Sakarya	Karasu	Yenimahalle	61
11	Antalya	Aksu	Isparta yolu	22	58	Sakarya	Kaynarca	Merkez	48
12	Antalya	Kemer	Goynuk	19	59	Kocaeli	Kandira	Agva yolu	51
13	Antalya	Kumluca	Kumluca	380	60	Istanbul	Agva	Kucukasagi	96
14	Antalya	Demre	Gurses	377	61	Istanbul	Sile	Merkez	16
15	Mugla	Fethiye	Gokben	503	62	Istanbul	Beykoz	Merkez	214
16	Mugla	Ula	Portakalli	77	63	Kocaeli	Golcuk	Basiskele	9
17	Mugla	Marmaris	Marmaris	53	64	Kocaeli	Karamursel	Kaytazdere	29
18	Mugla	Milas	Kemerkoy	23	65	Yalova	Merkez	Sogucak	29
19	Aydin	Kusadasi	Dilek Yarimadasi	59	66	Yalova	Cinarcik	Merkez	5
20	Izmir	Urla	Bademli	100	67	Bursa	Gemlik	Kursunlu	12
21	Izmir	Karaburun	Ambarseki	163	68	Bursa	Mudanya	Merkez	4
22	Giresun	Merkez	Mezarlik	2	69	Balikesir	Erdek	Ocaklar	4
23	Giresun	Bulancak	Kuçuklu	10	70	Balikesir	Bandirma	Edincik	200
24	Giresun	Eynesil	Cavuslu	10	71	Izmir	Selcuk	Sirince	339
25	Trabzon	Carsibasi	Buyukdere	170	72	Izmir	Tire	Baskoy	305
26	Rize	Pazar	Liman	5	73	Aydin	Merkez	Efeler	59
27	Trabzon	Yomra	Kasustu	129	74	Aydin	Sultanhisar	Salavatli	74
28	Trabzon	Merkez	Degirmendere	22	75	Balikesir	Ayvalik	Merkez	77
29	Ordu	Fatsa	Bolaman	5	76	Balikesir	Altinoluk	Merkez	11
30	Ordu	Merkez	Altinordu	5	77	Canakkale	Kucukkuyu	Mihli	7
31	Samsun	Merkez	Barincik	21	78	Canakkale	Merkez	Güzelyali	4
32	Samsun	Ondokuzmayis	Geleric	2	79	Canakkale	Lapseki	Merkez	19
33	Samsun	Bafra	Kelikler	8	80	Canakkale	Eceabat	Alcitepe	80
34	Samsun	Alacam	Yukarielma	15	81	Tekirdag	Merkez	Barbaros	15
35	Samsun	Alaca	Esentepe	249	82	Tekirdag	Sarkoy	Gazikoy	25
36	Samsun	Yakakent	Merkez	15	83	Tekirdag	Sarkoy	Murefte	11
37	Sinop	Gerze	Merkez	7	84	Tekirdag	Sarkoy	Merkez	11

38	Sinop	Erfelek	Merkez	169	85	Tekirdag	Merkez	Degirmenal ti	8
39	Sinop	Merkez	Osmaniye	115	86	Istanbul	Fatih	Gulhane	41
40	Sinop	Ayancik	Dervent	61	87	Istanbul	Adalar	Buyukada	3
41	Sinop	Turkeli	Merkez	30	88	Istanbul	Uskudar	Fethipasa	87
42	Kastamonu	Merkez	Ginolu	70	89	Bursa	Iznik	Hocakoy	185
43	Kastamon	Abana	Merkez	12	90	Yalova	Merkez	Elmalik	78
44	Kastamonu	Inebolu	Gemiciler	62	91	Bursa	Mustafak emalpasa	Akcapinar	39
45	Kastamonu	Doganyurt	Kayran	233	92	Izmir	Menemen	Etae	10
46	Kastamonu	Cide	Aydincik	145					
47	Kastamonu	Cide	Kuscu	16					
